# Phylogenetic relationships within the primitive acanthomorph fish genus *Polymixia*, with changes to species composition and geographic distributions

**DOI:** 10.1371/journal.pone.0212954

**Published:** 2019-03-01

**Authors:** W. Calvin Borden, Terry C. Grande, Mark V. H. Wilson

**Affiliations:** 1 Department of Biology, Saginaw Valley State University, University Center, Michigan, United States of America; 2 Department of Biology, Loyola University Chicago, Chicago, Illinois, United States of America; 3 Department of Biological Sciences, University of Alberta, Edmonton, Alberta, Canada; DePaul University, UNITED STATES

## Abstract

The genus *Polymixia* is the only survivor of a Late Cretaceous marine fish radiation and is often said to be the most primitive living acanthomorph (i.e., *Polymixia* possesses the greatest number of primitive character states for Acanthomorpha). Recent studies, including this one, place *Polymixia* as the sister to all other Paracanthopterygii. Despite its importance, most species of *Polymixia* are extremely difficult to discriminate on the basis of morphology. As a result, the number of valid species is uncertain. Moreover, there has never been a phylogenetic analysis of the genus. Thus, a molecular phylogenetic study was needed to clarify species boundaries and to resolve relationships within the genus. Tissue or DNA samples backed by museum vouchers were obtained for most species, with additional samples from new geographic areas representing specimens with distinctively different meristics and uncertain identifications. Seven loci (five nuclear and two mitochondrial) were sequenced, from which Bayesian and maximum-likelihood trees were generated. Results reveal nine species-level clades, of which five represent previously known species (*Polymixia berndti*, *P*. *japonica*, *P*. *longispina*, *P*. *lowei*, and *P*. *nobilis*). Surprisingly, results also reveal four previously unknown species-level clades, one close to *P*. *lowei*, one close to *P*. *nobilis*, and two new species clades related to *P*. *japonica*. The species clades are distinguished by their phylogenetic histories, sequence differences, geographic distributions, and morphologies. The clade containing *P*. *berndti* is recovered as the sister to all other species of *Polymixia*. Its genetic variability suggests that it might contain two or more species and it is referred to here as a “species complex”. *Polymixia nobilis*, the type species, was previously thought to be restricted to the Atlantic, but is now shown to be widespread in the Pacific and possibly in the Indian Ocean. Specimens from waters off Australia identified as *P*. *busakhini* actually belong to *P*. *nobilis*. In contrast, *P*. *japonica* is confirmed only in the area near Japan and the East China Sea; other more distant records are misidentifications. Wide (antipodal) geographic distributions are seen in several clades, including *P*. *nobilis*, the *P*. *berndti* species complex, and the *P*. *japonica* species group. The new phylogeny helps explain the evolution of some morphological characters previously used to distinguish groups of species, particularly dorsal-fin soft-ray count, shape of rows of scale ctenii, and number of pyloric caeca.

## Introduction

**Fig 1:**
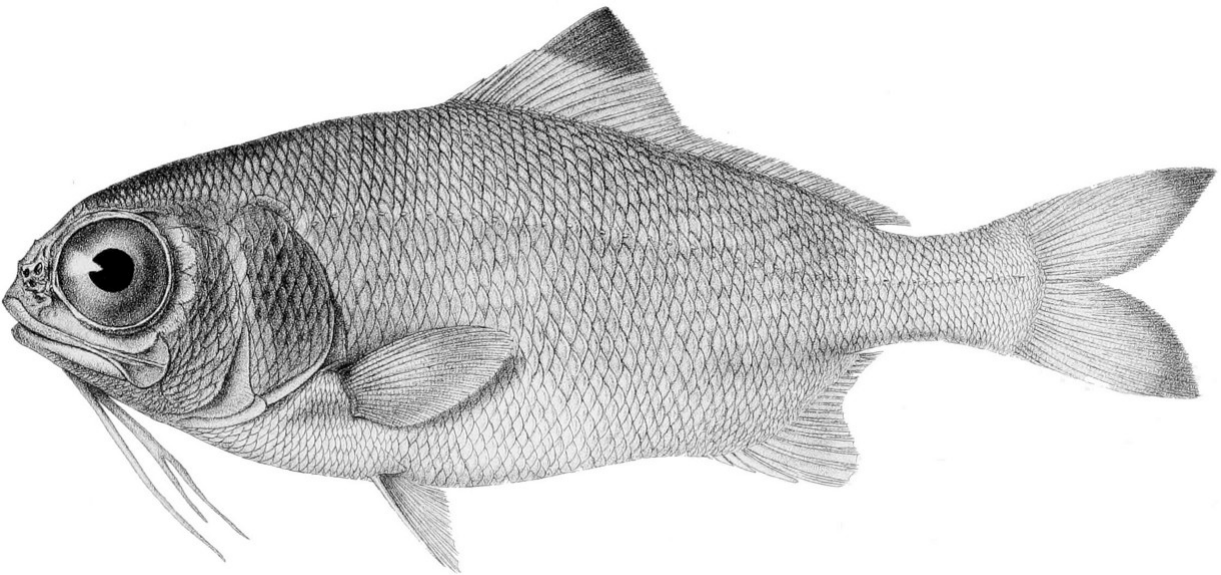
“*Polymixia nobilis*” as figured by Günther in 1887 [4]. In that paper, Günther placed his own [5] *P*. *japonica* Günther, 1877, in synonymy of *P*. *nobilis*. This drawing was based on a specimen collected off Inosima, Japan, the type area for *P*. *japonica*, and thus might represent *P*. *japonica* instead of *P*. *nobilis*. This public-domain illustration was accessed at https://commons.wikimedia.org/wiki/File:Polymixia_nobilis1.jpg and reversed left–right.

Members of the genus *Polymixia*, order Polymixiiformes, are known as the beardfishes for a prominent pair of hyoid barbels under the chin (Fig 1). *Polymixia* is the only surviving genus of a Late Cretaceous (Cenomanian; 100 million years ago) radiation [1, 2], and is thus considered a ‘living fossil’ or ‘relic.’ *Polymixia* has long been viewed as the key to understanding the origin and early radiation of acanthomorphs, or spiny-rayed teleosts, which today make up half of all living fish species and one quarter of all vertebrates. Patterson [3] wrote, “If there is an acanthomorph equivalent of the living monotremes amongst mammals, it is *Polymixia”*.

The higher-level classification of *Polymixia* has had a long and complicated history. Currently, the genus is usually placed in the order Polymixiiformes within the Paracanthopterygii, a group that also includes Percopsiformes, Zeiformes, Stylephoriformes, and Gadiformes [6]. However, for many years, *Polymixia* was thought to belong among the Beryciformes [7–12]. More recently, based on morphology, the paracanthopterygian affinities of *Polymixia* have been recognized, with placement either as sister to or as the basal (earliest branching) lineage within paracanthopterygians [2, 6, 13, 14]. Other morphological studies have placed it as sister to all other acanthomorphs [15–17], or as sister to acanthomorphs except lampriforms [1, 18–21], Molecular phylogenies have also provided disparate results, placing *Polymixia* as sister to the paracanthopterygians [22–24], sister to zeiforms plus acanthopterygians [25], sister to acanthopterygians [26], or sister to percopsiforms with both being either the basal acanthomorph clade [27] or the sister to non-percopsiform paracanthopterygians [28–32]. Most recently, Hughes et al. [33] also supported the first of the above molecular topologies when they recovered *Polymixia* as a paracanthopterygian, sister to all other members of that group, in agreement with Grande et al. [23] and the classification in Nelson et al. [6].

Skeletal characters common to all species of *Polymixia* that have influenced its proposed phylogenetic position [2, 23, 34] include the presence of antorbital and orbitosphenoid bones in the skull. The supraoccipital has a large crest thickened centrally and forms a long wedge separating the frontals for about 2/3 of their length. *Polymixia* exhibits a forward extension of the supratemporal fossa, subocular shelves on all infraorbitals, and a basisphenoid with descending process that fails to reach the parasphenoid. The hyomandibula has two condyles and the endopterygoid bears teeth. There are two supramaxillae, but the posterior one lacks a process overlapping the anterior one. The premaxilla has both ascending and articular processes. A ‘beryciform foramen’ is present in the anterior ceratohyal [19].

The postcranial skeleton is also unusual. *Polymixia* is known for having three sets of intermuscular bones [35]; a bone interpreted by Patterson & Johnson as the first epineural, for example, is uniquely enlarged and displaced ventrolaterally into the horizontal septum [19]. There are three widely spaced predorsal bones; haemal and neural spines with paddle-shaped ends are distinctive. There is a reduction of branchiostegal rays to seven pairs; however, the first three pairs are uniquely modified to form the skeletal support for the hyoid barbels. The subthoracic pelvic fins have 7–8 rays but lack spines. The first radial of the anal fin is distinctively enlarged. A full spine on preural centrum 2 (PU2) is present, and three epurals and six autogenous hypurals are found in all species (pers. obs. TG) [10, 14, 23, 34].

Earlier studies of morphology have frequently used anatomical features of *Polymixia* in comparative studies at higher taxonomic levels but have paid little attention to the relationships of and differences among its species. A notable exception was Kotlyar’s work on species diversity [12, 36], but even his studies were based mostly on external features and he did not attempt to understand the phylogenetic relationships among species. An underlying assumption in those works appears to have been that *P*. *nobilis* was not present in the Pacific, leading to naming of multiple new species, despite other authors having identified *P*. *nobilis* in the western Indian Ocean off Mozambique [37] and in the Coral Sea off Eastern Australia [38]. Our own review of the evidence revealed that many of the nominal species of *Polymixia* are so similar that morphological characters do not distinguish them reliably and are also too few for phylogenetic reconstruction.

Molecular systematists have often used samples of *Polymixia* in broad-scale phylogenetic analyses, but they have typically used very few samples from one or two species and have usually assumed that their samples were correctly identified. That assumption is examined critically in the present study.

### The species of *Polymixia*

Fourteen nominal species of *Polymixia* have been named; they are distributed in moderately deep waters in warmer regions of the Atlantic, Pacific, and Indian oceans and adjacent seas (about 45ºN to 45ºS). They are found at depths of about 100–700 m, over or near distal continental shelves, continental slopes, oceanic islands, and submarine seamounts. Of the 14 nominal species, 10 have usually been recognized as valid in recent works [6, 12].

All of the species are phenotypically very similar in body shape, color patterns, and most meristics (S1 Table); for example, common to all species are 29 (rarely 28) total vertebrae, five (rarely six) dorsal-fin spines, four anal-fin spines, and 7 (rarely 6) pelvic rays. The many similarities and the often-overlapping morphometric and meristic ranges [12] have made identifications and comparisons difficult. Except for *P*. *lowei* and *P*. *japonica*, the species are described solely on the basis of external morphology, meristics, counts of pyloric caeca, and geographic distributions. Some features of the skeletal morphology of *P*. *lowei* have been described [10, 14, 34], as have some skeletal features of *P*. *japonica* [39]. One or the other of these two species has also been used to represent the genus in many comparative and phylogenetic morphological studies (references above) as well as in numerous molecular phylogenetic studies [23, 26, 27, 40].

Earlier researchers also had difficulty distinguishing among species. Günther [4, 5] placed *P*. *japonica*, which he himself had named a decade earlier, in synonymy of *P*. *nobilis*; he was followed in this by some [41, 42], but not by later authors. Lachner [43] recognized *P*. *lowei* and *P*. *nobilis* as distinct but placed *P*. *berndti* in synonymy of *P*. *japonica*, whereas Yamane & Okamura [44] gave evidence that *P*. *berndti* is a valid species.

**Fig 2:**
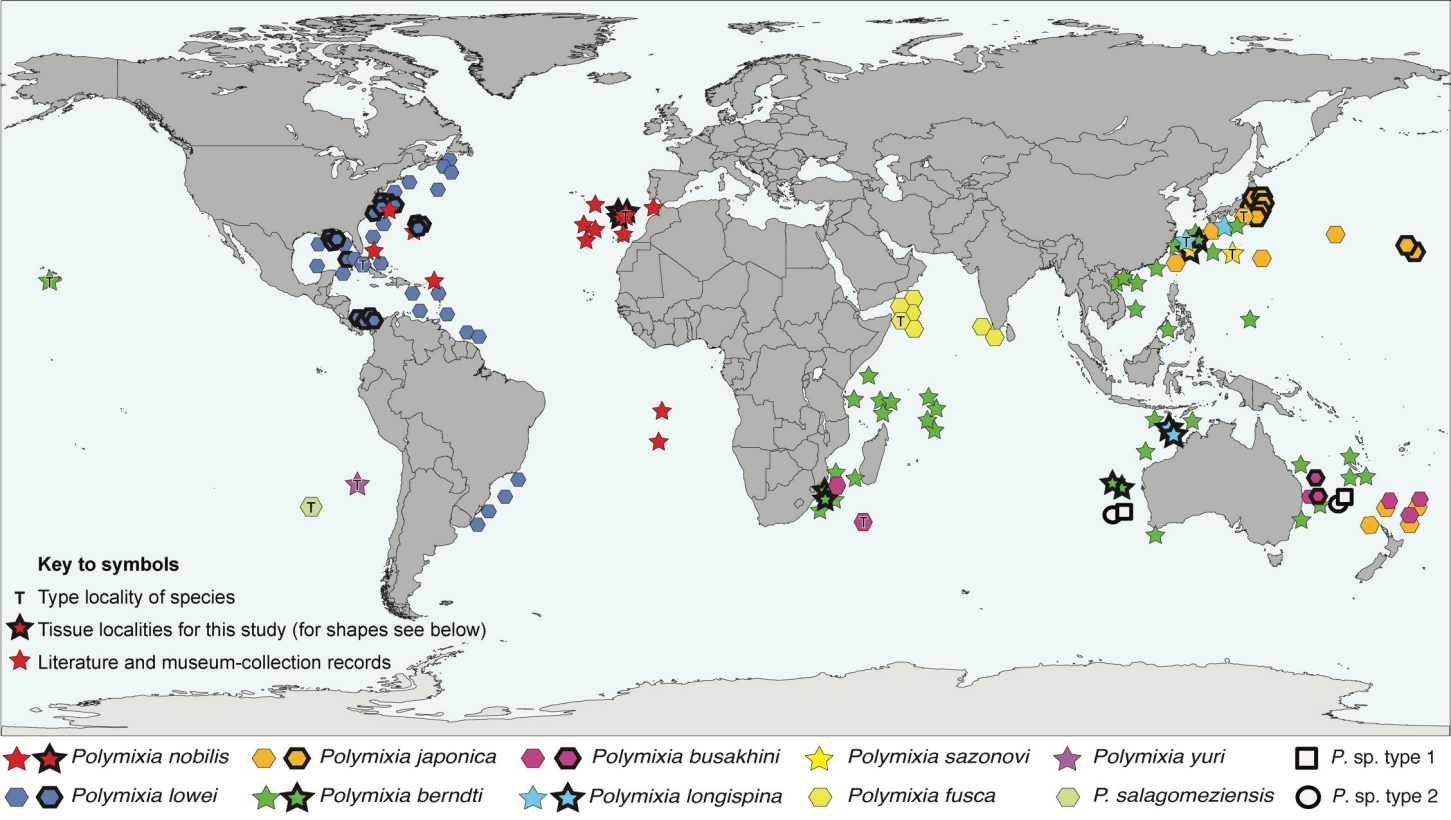
Global distribution of species of *Polymixia*. Type localities of species are indicated by symbols with an enclosed letter “T”. *Polymixia* sp. types 1 and 2 were considered by investigators to be unusual and not assigned to species. DNA sample localities for this study are indicated by symbols with bold outlines; see S2 Table for sample details. Global base map as modified by F. Bennet, in the public domain, accessed at https://commons.wikimedia.org/wiki/File:BlankMap-FlatWorld6.svg.

Because species of *Polymixia* appear so similar in most respects and their meristic and morphometric traits often overlap (S1 Table), researchers have given more weight than usual to geographic distribution patterns when identifying individuals or naming new species. It appears, for example, that *P*. *nobilis* was not seriously considered by some workers during identification of specimens from the Pacific Ocean. Distributions of the 10 species considered valid prior to our study are summarized below (by date of original description) and plotted in Fig 2. Each of the four nominal species considered to be a junior synonym is mentioned within the paragraph about its senior synonym.

#### Polymixia nobilis

Lowe, 1836, the type species of the genus, was based [45] on a specimen from Madeira in the Eastern North Atlantic. It has also been reported from, among other places, the northeastern Gulf of Mexico, off Virginia USA and from the Azores, Canary Islands, Bermuda, Cuba, the Bahamas, the Virgin Islands, and from the central South Atlantic near St. Helena [12, 46–48]. *Nemobrama webbii* Valenciennes, 1837, from the Canary Islands [49] and *Dinemus venustus* Poey, 1860, from off Cuba [50] are considered junior synonyms of *P*. *nobilis* [12].

#### Polymixia lowei

Günther, 1859, from the Western North Atlantic, was named [46] for a specimen from off Havana, Cuba. It has been reported from off southeastern Canada and eastern USA as well as from Bermuda, the northeastern Gulf of Mexico, and the southeastern Caribbean off Guiana [47]. *Polymixia nobilis virginica* Nichols & Firth, 1936, originally named [51] as a subspecies of *P*. *nobilis*, was placed in synonymy of *P*. *lowei* by Lachner [43].

#### Polymixia japonica

Günther, 1877, was based [5] on a specimen from off Inosima, Japan, and has since been reported from the East China Sea, New Caledonia, the Chesterfield Islands, the Hawaiian submarine seamount chain, and with some doubt as “*P*. cf. *japonica*” from off New Zealand [52]. Günther [4] placed *P*. *japonica* in synonymy of *P*. *nobilis*, but this was not followed by most authors [12, 43].

#### Polymixia berndti

Gilbert, 1905, was based [53] on a specimen from the Honolulu Fish Market, Oahu Island, Hawaii. The species has also been reported from waters off Mozambique and South Africa, off both Eastern and Western Australia, and from near Taiwan [12].

#### Polymixia fusca

Kotthaus, 1970, was named [54] for specimens from the northwestern part of the Indian Ocean near Somalia. It has also been reported off Yemen and off the southern tip of India and Sri Lanka [12].

#### Polymixia yuri

Kotlyar, 1982, and ***P*. *salagomeziensis*** Kotlyar, 1991, are both [55, 56] based on specimens from restricted eastern South Pacific areas west of Chile. *Polymixia yuri* is from the Naska submarine mountain ridge, and *P*. *salagomeziensis* is from the Sala y Gomes submarine ridge closer to Chile.

#### Polymixia longispina

Deng, Xiong & Zhan, 1983, was based [57] on specimens from the East China Sea; it has also been reported from near Taiwan and from waters off northwestern Australia. *Polymixia kawadae* Okamura & Ema, 1985, named [58] for specimens from off southern Japan, is now considered [12, 59] a junior synonym of *P*. *longispina*.

#### Polymixia busakhini

Kotlyar, 1992, was named [60] for a specimen from a submarine ridge 800 km south of Madagascar, with paratypes from waters off Eastern Australia. *Polymixia* specimens from both regions had earlier been reported as *P*. *nobilis* [37, 38]. The species has also been recorded from near Mozambique [12] and with doubt as “*P*. cf. *busakhini*” from submarine ridges north of New Zealand [52].

#### Polymixia sazonovi

Kotlyar, 1992, was based [12] on specimens taken near the Kyushu-Palau ridge in the western North Pacific, south of Japan.

Specimens of most nominal species of *Polymixia* are rare in collections and relatively little is known about their biology. Some (e.g., *P*. *nobilis*, *P*. *lowei*, *P*. *berndti*) appear to be sufficiently abundant that they are not likely to be endangered. However, *P*. *nobilis* is the only species listed by the IUCN Red List where its status is given as “Least Concern” [48]. For other species, there is a lack of information. Several of the nominal species are known only by type specimens from a single locality, while for others a precise locality is unknown because type or other museum specimens have been obtained in local fish markets, e.g., in Japan–*P*. *kawadae* [58], Hawaiian Islands–*P*. *berndti* [53], and Madeira–*P*. *nobilis* [9].

Concerning life history of the various species, not much is known [12]. For example, Moore [47] stated that some individuals had been observed swimming near the bottom with barbels in contact with the sediment. Kotlyar [61] reported that *P*. *berndti* in the Indian Ocean grew slowly and fed on crustaceans, small fishes, and squid. Woods & Sonoda [9] mentioned that *P*. *lowei* was found in stomach contents of the flounder *Paralichthys dentatus*, and Heemstra et al. [62] reported that *P*. *berndti* was included as a prey item of the coelacanth, *Latimeria chalumnae*. Evidently there is still much to learn about the biology and life history of members of this genus.

Because of the difficulty in identifying species of *Polymixia* based on morphology, a different approach to understanding the species composition within the genus was needed. Moreover, no previous study using any source of data has attempted a phylogenetic analysis of the species of *Polymixia*. Earlier work [12, 36] aligned particular species based on similarities in individual characters. The present study assesses both the species composition and the phylogenetic interrelationships of *Polymixia* using a novel molecular data set in which almost all of the nuclear loci are sequenced for the first time. With a revised species composition and a new phylogeny, we are able to re-evaluate the significance of the earlier proposed morphological characters. We are also able to correct a number of misidentifications including some based on DNA barcodes, and to reassess and propose revised geographic ranges for many species. Results of this study should help our understanding of which species are at greatest risk of extinction, give a firmer basis for future studies of comparative biology and morphology within the genus, and provide a more accurate interpretation of evolutionary changes among closely related groups of acanthomorphs.

## Materials and methods

### Taxon sampling

Tissue samples and DNA extractions were obtained from either museum or researcher collections, nearly all backed by voucher specimens. We amassed 47 samples of *Polymixia* (S2 Table) identified to seven of the 10 beardfish species currently considered valid (genetic samples for *P*. *fusca*, *P*. *salagomeziensis*, and *P*. *yuri* were not available) and five problematic samples identified only to genus. Samples collectively had a cosmopolitan marine distribution except for the polar seas (Fig 2). Samples for eighteen outgroups (S2 Table) represented the percopsiforms (n = 3), gadiforms (n = 2), stylephoriform (n = 1), zeiforms (n = 3), aulopiforms (n = 1), myctophiforms (n = 1), lampriforms (n = 2), trachichthyiforms (n = 1), holocentriforms (n = 1), beryciforms (n = 1), and percomorphs (n = 2).

### Laboratory methods

Genomic DNA was extracted from muscle tissues using the DNeasy Blood and Tissue Kit (Qiagen). Two mitochondrial fragments—*12S* [63, 64]; *16S* [65, 66]—and five single-copy nuclear DNA loci—*glyt*, *myh6*, *plagl2*, *ptr*, *sh3px3* [67]—were amplified using primers and PCR regimes in recent studies [23, 67–69]. The nuclear DNA loci were amplified using nested primers in which product from the first PCR served as template in a subsequent PCR with internal primers relative to the original amplicon [67]. Amplicons were sequenced using Sanger sequencing methods by the DNA Analysis Facility on Science Hill at Yale University, New Haven, CT.

### Sequence assembly and alignment

Sequences were compiled into contigs, edited, and aligned initially using Geneious v.11.1.4 (Biomatters Ltd.). Nuclear DNA loci were aligned by amino acid after translation. When indels were present (*glyt*, *myh6*, *plagl2*) the alignment was adjusted by eye. Our *12S* amplicons included a small portion of the 3’ end of *12S*, *tRNA-Val*, and the 5’ end of *16S*. The *16S* portion was the majority of the amplicon, and we elected to use only this portion. Our *16S* amplicons were composed entirely of *16S* and covered a region toward its 3’ end. The two *16S* fragments were not contiguous [70]. Both fragments were aligned using an online version of MAFFT v7 [71, 72].

### Partitioning

PartitionFinder v2.1.1 [73] inferred the optimal partitioning scheme from a greedy search algorithm [74] using both AICc and BIC criteria implemented by PhyML [75]. In cases where the partition was identified by “invariant and gamma” substitution rates (“I+G”), the rates model in analyses was reduced to “gamma” (“G”).

### Matrix characteristics

The sole tissue originally identified as *P*. *sazonovi* failed to amplify or sequence for every locus, leaving a matrix with 64 OTU’s, 46 of which had been identified previously to 6 nominal species of *Polymixia* or to *Polymixia* sp. The five nuclear DNA loci totaled 3790 base pairs (*glyt*–864 bp, *myh6*–780, *plagl2*–740, *ptr*–679, *sh3px3*–727), the alignment of which did not vary across analyses. The nuclear DNA matrix was 95% complete with missing sequences from *plagl2* (6 missing) and *ptr* (8 missing) loci; outgroups accounted for five of the 14 missing sequences (all *ptr)*. For the two mitochondrial DNA loci (i.e., both *16S* fragments; 1193 bp), the matrix was 96% complete, with only five sequences missing, two of the five being in outgroup taxa. GenBank Accession numbers for submitted sequences and those used for outgroups are listed in S2 Table.

The mitochondrial DNA (*16S*) alignments determined by E-INS-i (1200 bp), Q-INS-i (1237 bp), and Auto (1193 bp) strategies in MAFFT varied slightly by length and location of indels. The mitochondrial DNA alignment used for our Bayesian and maximum-likelihood analyses is the one determined by the “Auto” option in MAFFT, and when combined with nuclear DNA loci, we refer to it as the ‘Auto-matrix’ (S1 File).

Three and four partitions were identified using BIC and AICc, respectively. In BIC, first and second-position codons of nuclear DNA loci were modeled by K81UF+I+G, third-position codons by TVM+G, and *16S* fragments by GTR+I+G. AICc identified first and second-position codons modeled by GTR+I+G; third-position codons of *glyt* and *myh6* by TVM+G; third-position codons of *plagl2*, *ptr*, and *sh3px3* by GTR+G; and *16S* fragments by GTR+I+G.

All Bayesian analyses converged as indicated by mean standard deviations of split frequencies (< 0.01), PSRF values of 1.0, and “nruns” of 2. A relative burn-in of 25% was sufficient and applied to all Bayesian analyses.

### Phylogenetic analyses

Analyses were performed using Bayesian inference and two different maximum-likelihood algorithms. The effects on tree topologies of (1) Bayesian versus ML algorithms on the same matrix, (2) AICc versus BIC-based partition schemes on the same matrix, and (3) different mitochondrial DNA alignments were explored.

Bayesian analyses used MrBayes v3.2.6 [76] conducted for 25,000,000 iterations with a sampling frequency of 500 for 2 runs and 4 chains using a Metropolis-coupled MCMC [77]. To minimize the possibility that runs would be trapped in local minima, the mean exponential prior on branch lengths was decreased to 0.01 [78, 79]. Variations of the GTR rate matrix (e.g. TVM) were converted to the “GTR” model. Bayesian analyses were evaluated for convergence by the mean standard deviation of split frequencies and Potential Scale Reduction Factors (PSRF) of MrBayes. A 25% relative burn-in was confirmed by viewing log likelihood values in Tracer v1.6.1 [80]. Nodal support was estimated using posterior probabilities on a calculated consensus tree, which was viewed with FigTree v1.4.3 [81]. Maximum-likelihood (ML) analyses using Garli v2.0 [82, 83] employed two runs of 100 search replicates with stepwise addition. Nodal support was estimated using a nonparametric bootstrap from 1000 pseudoreplicates. Maximum- likelihood analyses using RAxML v8.2.12 [84] were performed for 100 searches beginning with a random tree and a GTRGAMMA model of nucleotide substitution for each partition. Nodal support was estimated from 1000 bootstrap replicates and mapped onto the best-known ML tree from the 100 searches.

### Assessment of species boundaries

Distinct, monophyletic groups identified in the phylogenetic analyses represent possible species according to the Phylogenetic Species Concept [85]. We evaluated evidence that each of these clades represents a distinct and diagnosable evolutionary species by examining phylogenetic relationships, genetic distinctness, geographic distributions, and meristic and morphological evidence for characteristic features.

Phylogenetic evidence was assessed by comparing the monophyly and relationships of the possible species clades in trees generated by different partition models, alignments, and phylogenetic algorithms.

Genetic distinctness was assessed through sequence differences within and among species clades based on the multi-locus alignment in this study, and by comparison with clusters of “barcode” sequences using the Neighbor-Joining identification tree from BOLD Systems [86] for the genus *Polymixia*. The BOLD results are independent of ours because our matrix did not include sequence from the mitochondrial DNA COI gene. BOLD results are primarily useful for assigning specimens to putative species or making preliminary identifications. The public database contained data for only a subset of the relevant species clades or clusters; we therefore compared our results with the identification tree that used the entire BOLD database, including both public and private or restricted records. For some records, geographic locality and voucher identifications were private and unavailable to us; for many other BOLD records there was published voucher information, and for still others we could recognize which voucher specimens were the tissue sources (details for key examples are presented below in Results). BOLD results also provided additional records that were used to help revise geographic distributions, after correcting for mis-identified samples.

Morphological evidence was assessed by examining characters previously considered important by Kotlyar [12] for distinguishing species of *Polymixia*. Kotlyar’s data were also compared to new observations using alcohol-preserved and cleared-and-stained specimens (see Appendix) as well as data generously provided by J. Pogonoski of the Australian National Fish Collection, CSIRO. Some of the more important characters were mapped onto our resulting phylogeny to examine character-state changes on certain lineages.

## Results

### Phylogenetic results

**Fig 3:**
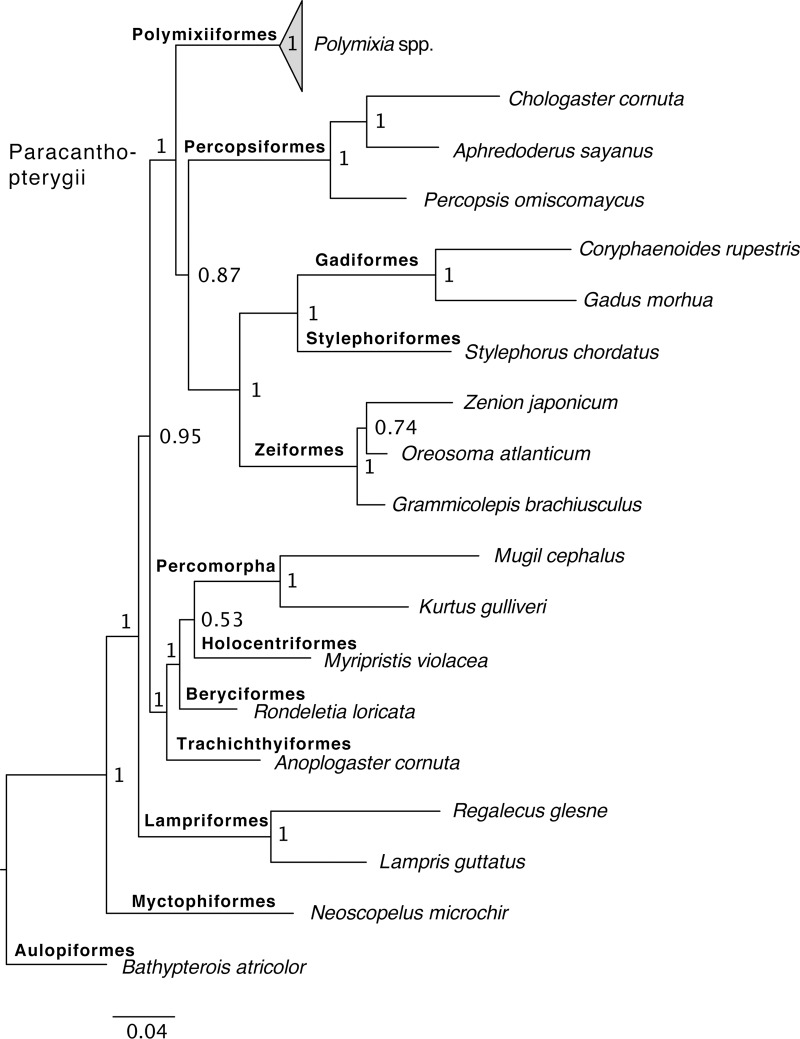
Bayesian phylogeny of Polymixiiformes and outgroups. The tree is based on analysis of the combined molecular matrix using MrBayes [76] with the BIC partition scheme of Partition Finder [73] and Auto alignment of mitochondrial DNA loci in MAFFT [72]. Sequence sources and sample numbers are given in S2 Table.

The Bayesian analysis and both of the maximum-likelihood analyses produced almost identical trees, with *Polymixia* as the basal (earliest-branching) clade within the Paracanthopterygii, as seen in the outgroup relationships of Fig 3.

**Fig 4:**
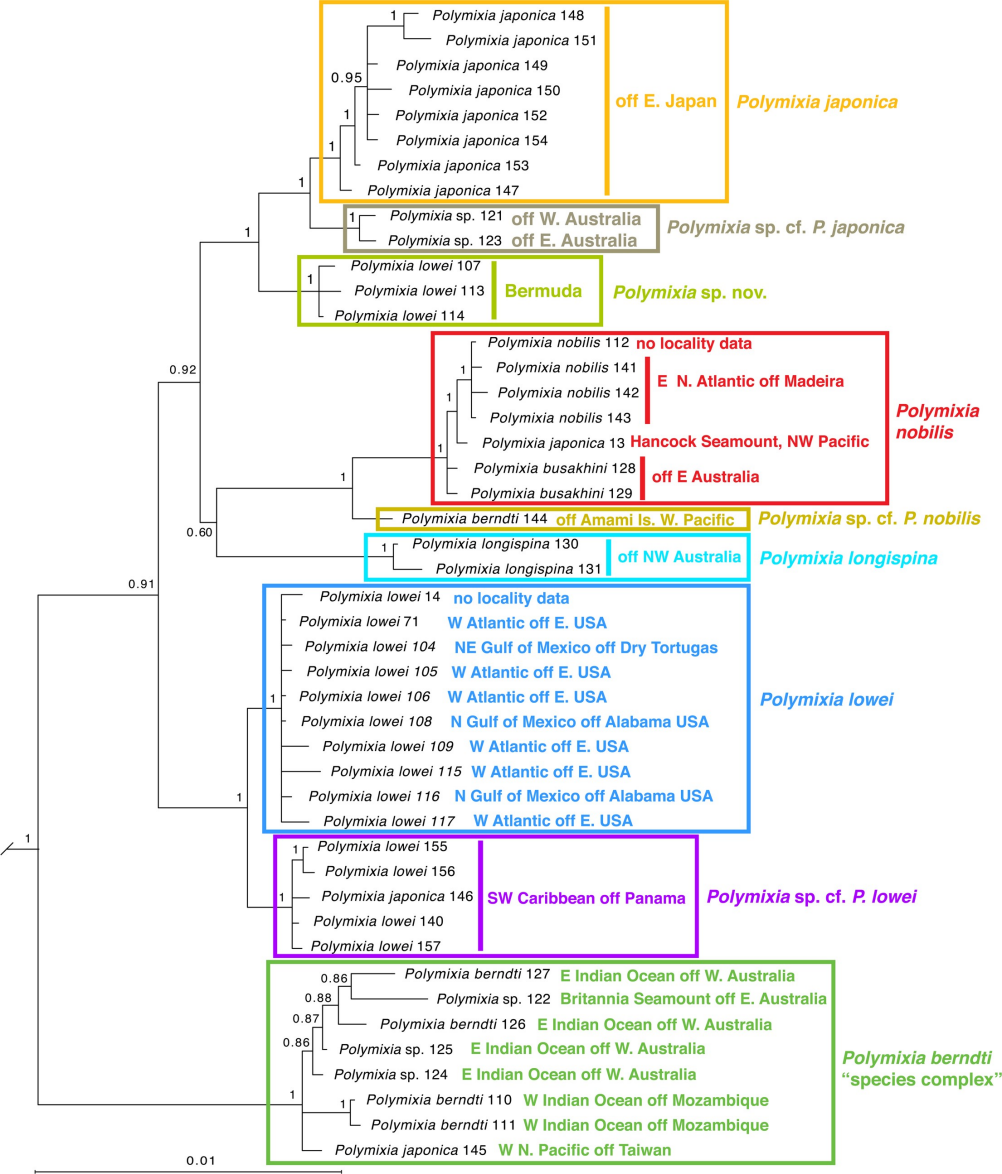
Bayesian phylogeny of the genus *Polymixia*. The analysis used MrBayes [76] with the BIC partition scheme of Partition Finder [73] and Auto alignment of mitochondrial DNA loci in MAFFT [72]. For outgroup relationships see Fig 3. The nine species clades are indicated by colored rectangles. Ingroup samples are shown with their original identifications, sample numbers, and approximate geographic locations. Sequence sources and sample numbers are given in S2 Table.

**Fig 5:**
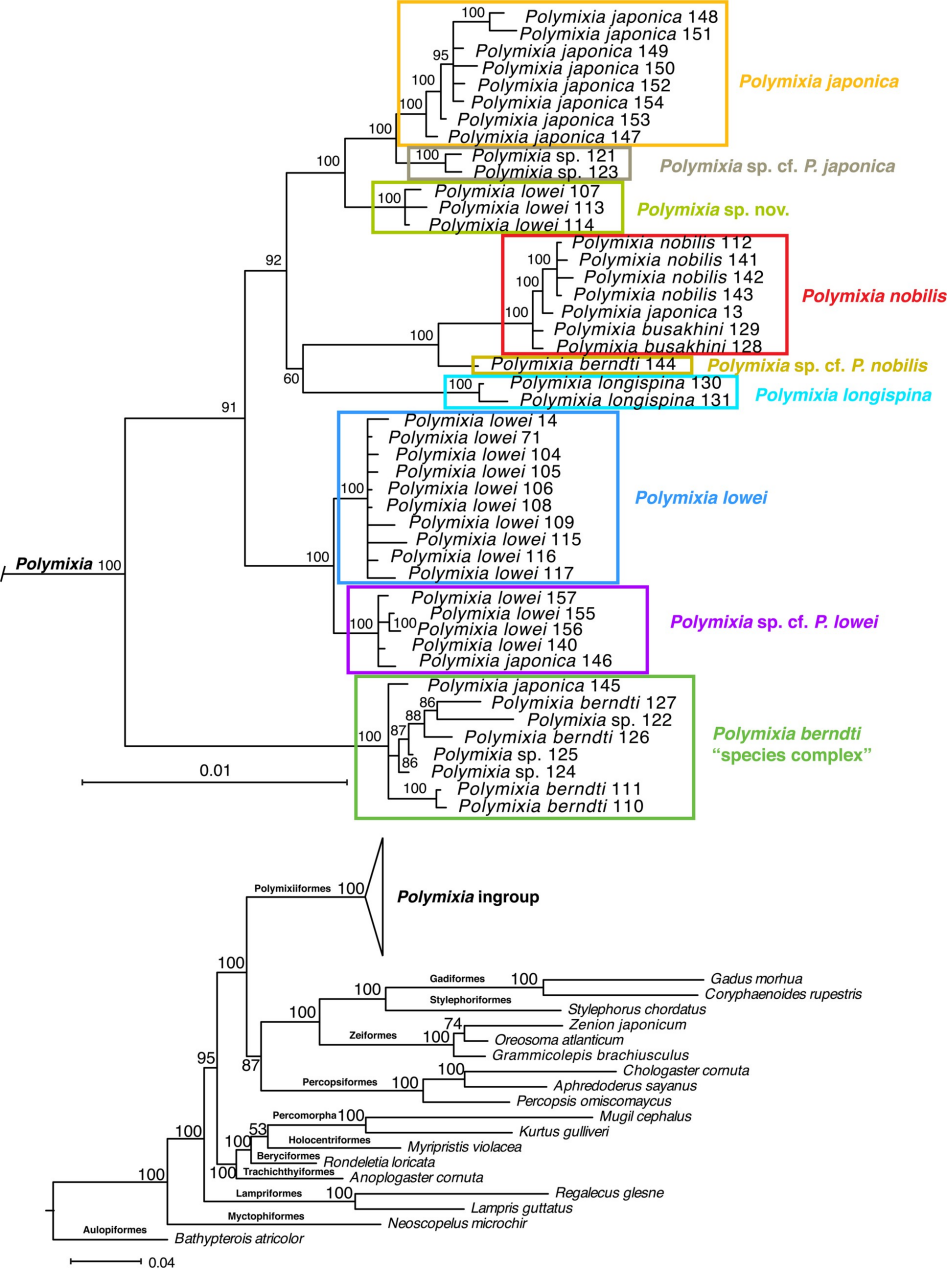
Maximum-likelihood phylogeny of the genus *Polymixia* using Garli. This analysis used Garli v2.0 [83] with the BIC partition scheme of Partition Finder [73] and Auto alignment of mitochondrial DNA loci in MAFFT [72]. The nine species clades are indicated by colored rectangles. Ingroup samples are shown with their original identifications, sample numbers, and approximate geographic locations. Sequence sources and sample numbers are given in S2 Table.

**Fig 6:**
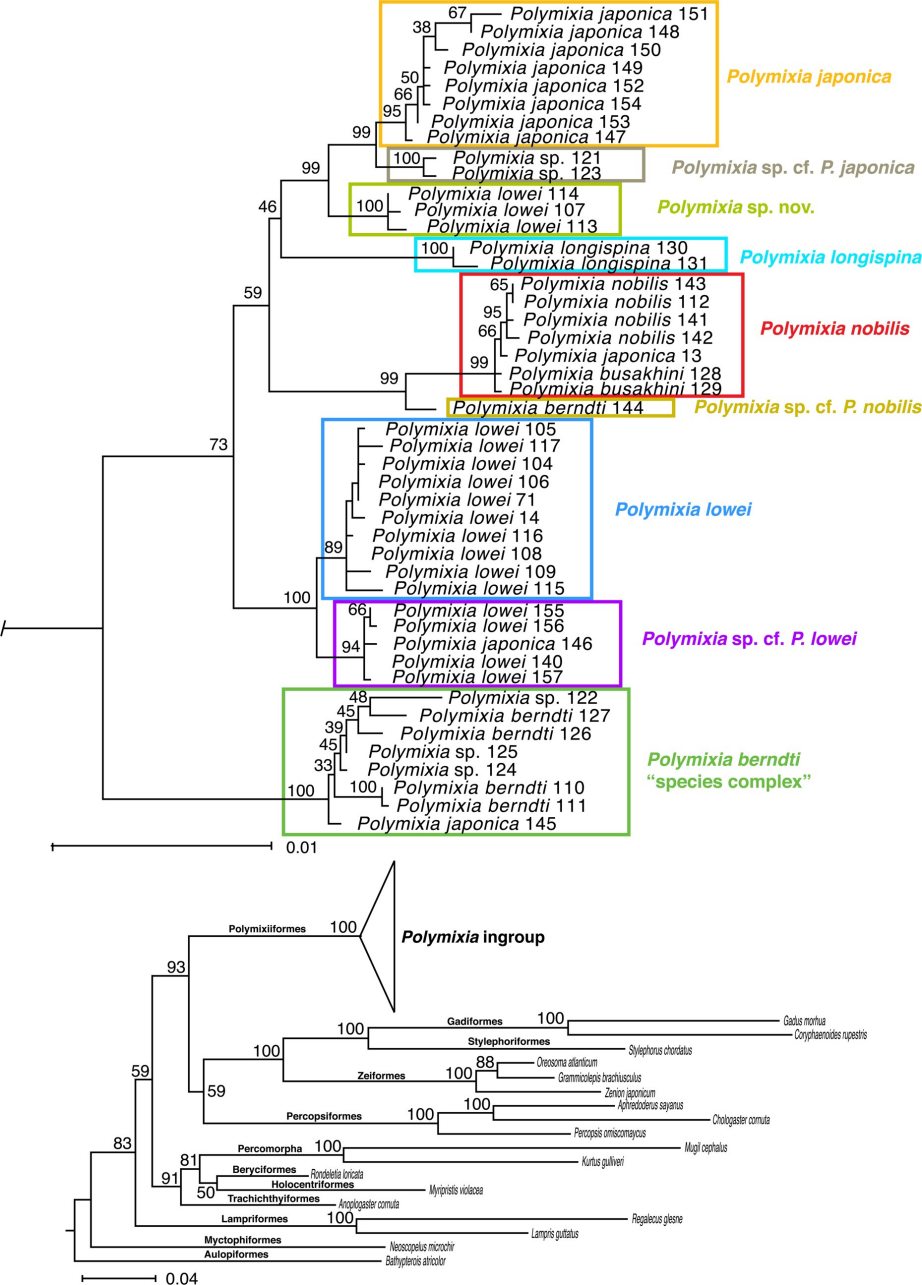
Maximum-likelihood phylogeny of the genus *Polymixia* using RAxML. This analysis used RAxML v8.2.12 [84] with the BIC partition scheme of Partition Finder [73] and Auto alignment of mitochondrial DNA loci in MAFFT [72]. The nine species clades are indicated by colored rectangles. Ingroup samples are shown with their original identifications, sample numbers, and approximate geographic locations. Sequence sources and sample numbers are given in S2 Table.

Within *Polymixia*, our results resolve five distinct groups of species (Figs 4–6): three species (*P*. *japonica*, *Polymixia* sp. cf. *P*. *japonica*, and *Polymixia* sp. nov.) in the *P*. *japonica* species group, two species (*P*. *nobilis* and *Polymixia* cf. *P*. *nobilis*) in the *P*. *nobilis* species group, *P*. *longispina* as its own group, two species (*P*. *lowei* and *Polymixia* cf. *P*. *lowei*) in the *P*. *lowei* group, and at least one in the *P*. *berndti* “species complex”. All analyses place the *P*. *berndti* complex as sister to all other species, and among the other species, the *P*. *lowei* species group is sister to the remaining species.

The Bayesian (Figs 3 and 4) and Garli ML (Fig 5) topologies agree in all important details, while the RAxML topology (Fig 6) has a minor rearrangement in the outgroups (*Myripristis* sister to *Rondeletia* versus these genera as sequential branches) and a minor ingroup rearrangement in the location of *P*. *longispina* (weakly related to the *P*. *japonica* group in RAxML versus weakly related to the *P*. *nobilis* group in Bayesian and in Garli ML).

All three analyses identified nine distinct clades, which we recognize as different species (five with available names and four apparently unnamed); the nine clades have identical memberships in the different analyses and, except as mentioned for RAxML, identical relationships to their closest relatives. Changing the partition schemes (e.g., using AICc) had no bearing on outgroup or ingroup relationships. Outgroup relationships and memberships of the nine ingroup clades were well supported as estimated by Bayesian posterior probabilities (Figs 3 and 4). Some detailed relationships among samples within each ingroup clade had lower posterior probabilities, but this is expected for samples that are very similar genetically. These patterns were seen also in the ML results, with the most important of the bootstrap values in the Garli tree at 90–100% (Fig 5) and only slightly lower values in the RAxML tree (Fig 6).

Different alignment algorithms (E-INS-i, Q-INS-i, Auto) for the two 16S loci, when analyzed together with nuclear DNA loci in MrBayes, yielded nearly identical results for E-INS-i and Auto and only minor differences in topology (e.g., relationship of *Rondeletia* to *Myripristis*) in the tree based on the Q-INS-i alignment.

**Fig 7:**
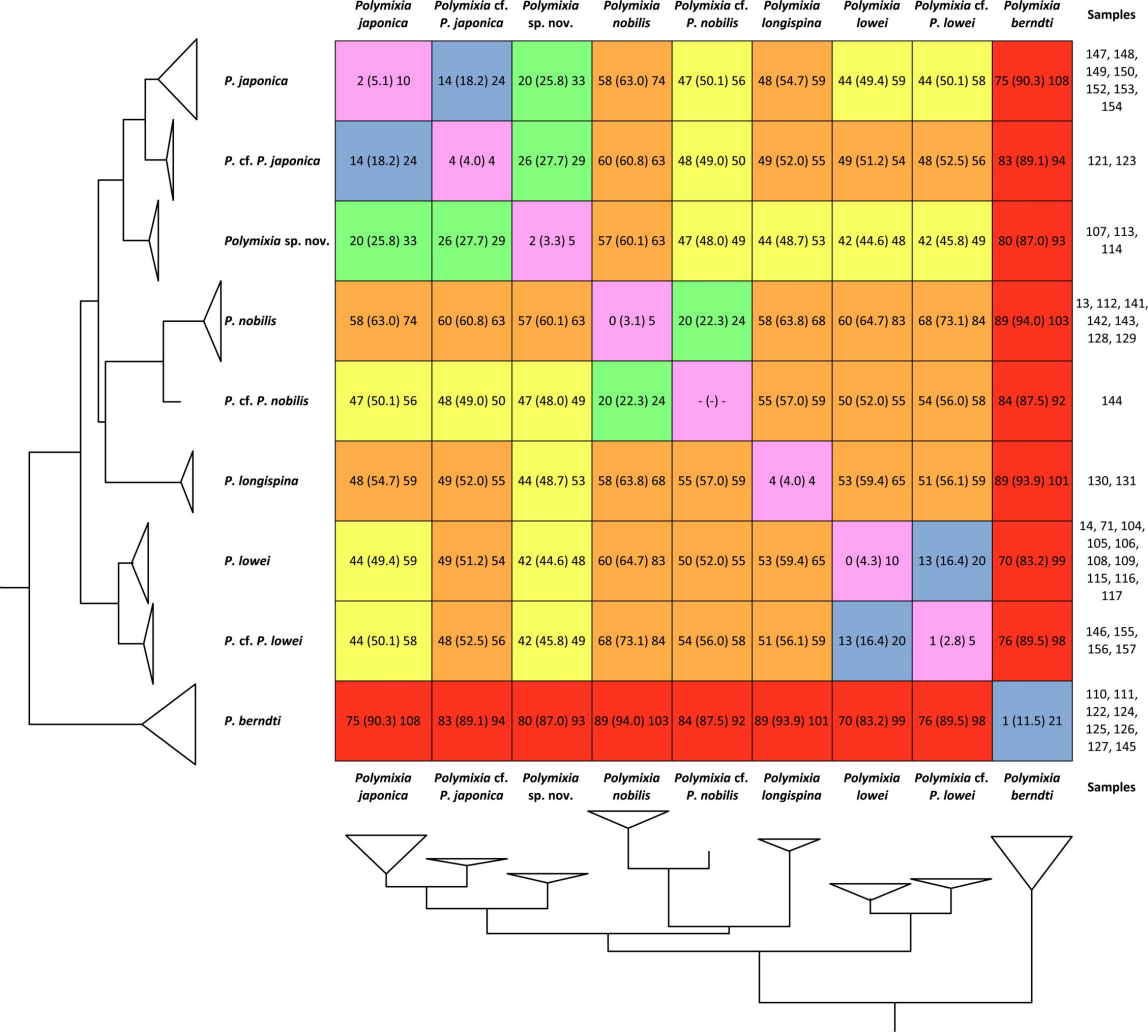
Sequence differences among and within species clades of *Polymixia*. The sequence difference between each pair of species is the total number of sites with different nucleotides based on our alignment of 4983 sites (S1 File); comparisons are given as observed range with mean in parentheses. Colors indicate mean number of differences out of 4983 as follows: 0–10, purple; 11–20, blue; 21–30, green; 41–50, yellow; 51–80, orange; 81–100, red.

With one exception, clades contain samples that differ from one another by no more than 10 sites over the 4983 positions in the alignment (Fig 7). The *P*. *berndti* clade is the exception as it contains samples differing by between 1 and 21 bases out of 4983, a range about twice that within other species clades. We suggest that this clade represents a species complex in which there is unrecognized species diversity.

As expected, samples belonging to closely related species clades in the phylogeny (Figs 4–6) have fewer sequence differences than those between more distantly related clades (Fig 7). Examples include sequence differences between the single sample of *Polymixia* cf. *P*. *nobilis* and samples of *P*. *nobilis* itself (20–24), samples of *Polymixia* cf. *P*. *japonica* and *P*. *japonica* itself (26–29) and between *Polymixia* sp. nov. and *P*. *japonica* itself (20–33).

Among individual specimens, differences vary from 0 to 108 nucleotide differences over the 4983 bp in our alignment. Average differences between all pairs of species clades vary from 20 to 94 (Fig 7), a range in percentage terms of 0.40% to 1.89%. The loci in our phylogenetic analyses are thus far more conservative than the 648 bp of sequence from the 5’ end of the COI mitochondrial gene used in BOLD [86], for which differences among species clusters within *Polymixia* vary from about 2% to about 15% (S1 Fig).

Comparison of the species clades in our analyses with the clusters generated by the NJ (Neighbor Joining) tree in the species identification function of BOLD shows that most of the clusters we identified using multiple loci can also be detected in the BOLD i.d. tree (S1 Fig), provided that all BOLD records of the genus *Polymixia* are included, both those with public and those with private data. However, the BOLD data also include many conflicts of species identifications, potentially leading to erroneous conclusions about the identity of some BOLD clusters, and a cluster “tree” that differs in topology from our phylogenetic trees. The correct identities of the BOLD clusters are discussed below.

Results specific to each species group and its contained species clade(s) are presented in the following sections:

#### *Polymixia berndti* species complex

***Polymixia berndti*** was named for a specimen from off Honolulu [53]. Specialists usually have been able to identify specimens of *P*. *berndti* based on morphological characters, as judged by specimens consistently assigned to the clade in our results; however, an exception is one sample originally from off Amami Island in the southern Japanese Archipelago, originally identified as *P*. *berndti* but re-identified herein as *Polymixia* sp. cf. *P*. *nobilis* (see below). Lack of genetic material from the Hawaiian Islands type area means that there remains a small amount of doubt that the DNA-based clade recovered here is conspecific with *P*. *berndti*.

Samples of *Polymixia berndti* form a clade that is the most basal (i.e., earliest branching, and sister to all other species) clade within *Polymixia*. This clade is also the most distinct genetically in the genus, with sequence differences ranging from a low of 70–99 versus *P*. *lowei* to a high of 89–103 versus *P*. *nobilis* (Fig 7). These samples are from the western Indian Ocean off Mozambique, the eastern Indian Ocean off Western Australia, the southwestern Pacific (Coral Sea) off Eastern Australia, and the Western Pacific (East China Sea) off Taiwan (Fig 2). Including the type area for this species in the Hawaiian Islands, the geographic distribution is nearly antipodal.

Genetic variation within this clade is about twice that in other species-level clades (1–21 nucleotide differences within the clade versus 0–10 within others). This and the long branches leading to some samples or pairs of samples suggest that future study with more specimens is needed to clarify whether *P*. *berndti* should be divided into more than one species.

#### *Polymixia japonica* species group

This species group is a clade containing three species, *Polymixia japonica*, *Polymixia* sp. cf. *P*. *japonica*, and *Polymixia* sp. nov.

***Polymixia japonica*** itself is represented in our study only from the Western Pacific off Japan, China, and Taiwan. All other reports that we have seen and for which there are genetic data (from Australia, the East China Sea, the Hawaiian seamount chain, and the Caribbean) are misidentifications of specimens of *P*. *berndti*, *P*. *longispina*, *P*. *nobilis*, and *P*. sp. cf. *P*. *lowei*, respectively (Figs 4–6). These include two samples from Hancock Seamount in the NW part of the Hawaiian submarine seamount chain; the original two samples proved to have identical sequences and have since been confirmed to be from a single fish specimen, which we can now re-identify as a specimen of *P*. *nobilis*. A single specimen from the Caribbean identified as *P*. *japonica* is here re-identified as *Polymixia* sp. cf. *P*. *lowei*. One sample from Taiwan identified as *P*. *japonica* is here re-identified as *P*. *berndti*. In the BOLD i.d. tree, one additional sample labeled *P*. *japonica* should be re-identified as *P*. *longispina*.

The only samples in our analyses that truly represent *P*. *japonica* (Figs 4–6) are all from waters off Japan. A corresponding cluster of only three samples in the BOLD i.d. tree (S1 Fig) can be recognized by also having closest similarities to two other clusters (*P*. sp. cf. *P*. *japonica* and *Polymixia* sp. nov. as identified herein). Two of the three BOLD samples of *P*. *japonica* have locality indications from the East China Sea off China and Taiwan, while the third has no public locality information. The distribution of confirmed *P*. *japonica* is thus greatly reduced from its previously assumed geographic range.

***Polymixia* sp. cf. *P*. *japonica*** is represented by two samples, one from off Eastern Australia and one from off Western Australia. They are distinct from the *P*. *japonica* clade but are most closely related to that species (Figs 4–6). The two samples have nucleotide differences at 14–24 sites (average 18.2) when compared with the eight samples of *P*. *japonica*. Despite being from different oceans (SW Pacific/Coral Sea and E Indian Ocean), the two are genetically very similar, differing from each other only at four sites out of 4983. A comparable cluster, potentially the same two individuals, is seen in the BOLD i.d. tree for *Polymixia* (S1 Fig).

***Polymixia* sp. nov.** is represented by three samples from Bermuda originally identified as *P*. *lowei* (Figs 4–6). However, the three samples belong neither to *P*. *lowei* nor to the only other species, *P*. *nobilis*, previously identified from that area [47, 48, 87]. We have located and examined voucher specimens for two of the three genetic samples of the new species (BAMZ lot 1997-159-006) and we have also examined preserved specimens of the other two species of *Polymixia* from Bermuda. We can thus confirm that *P*. *lowei* (e.g., BAMZ 1984-047-006, 1989-047-003) and *P*. *nobilis* (ANSP 124292) both also occur in Bermuda waters, though from different precise localities, as previously reported [87]. The Bermuda clade of *Polymixia* sp. nov. is sister to (*P*. *japonica* + *P*. sp. cf. *P*. *japonica*). The new Bermuda clade is genetically distinct from samples of *P*. *japonica* (20–33 nucleotide differences) and from *P*. sp. cf. *P*. *japonica* (26–29 differences). It represents a new, previously unsuspected species; because its existence was not recognized morphologically, it is a true cryptic species. A formal description of this new species is in preparation.

Although the geographic range of *P*. *japonica* has been reduced greatly by our results, the species of the *P*. *japonica* species group taken together are found on opposite sides of the globe: the antipode of Bermuda is in the Indian Ocean off Perth, Australia, near where one of the two specimens of *Polymixia* sp. cf. *P*. *japonica* was collected.

#### Polymixia longispina

The type locality of *P*. *longispina* is in the East China Sea [57]. Two samples previously identified as *P*. *longispina*, both from the eastern Indian Ocean NW of Australia, form a distinct clade that is not closely related to any other species of *Polymixia*. The two specimens differ from each other at only four out of 4983 nucleotide sites. A comparable cluster in the BOLD i.d. tree includes two additional samples, but their locality data are not public.

In Bayesian and Garli ML analyses, *P*. *longispina* is weakly grouped with the *P*. *nobilis* species group (Figs 4 and 5), but in the RAxML analysis it groups weakly with the *P*. *japonica* species group (Fig 6). It is about equally and markedly distinct (44–68 differences) in our data from all species of *Polymixia* except *P*. *berndti*, from which it is most distinct (89–101 differences). BOLD i.d. cluster results (S1 Fig) suggest a slightly greater sequence similarity for the COI barcode sequence to the *P*. *lowei* species group than to either the *P*. *japonica* species group or the *P*. *nobilis* group. Although this species is rare and difficult to place in the phylogeny, it is not the most difficult to identify from specimens because of its distinctively enlarged anal-fin spine and is treated here as a separate ‘species group’ of one species.

#### *Polymixia lowei* species group

*Polymixia lowei* and *Polymixia* sp. cf. *P*. *lowei* are sister species composed of specimens formerly identified as *P*. *lowei*. The circumscribed *P*. *lowei* and its unnamed sister species represent a sibling species pair. In our Bayesian results (Fig 4) and in our maximum-likelihood results (Figs 5 and 6), *P*. *lowei* and its sibling species are the sister group to all *Polymixia* except the *P*. *berndti* “species complex”.

***Polymixia lowei*** has been reported from its type area near Havana, Cuba [46], as well as from the Western Atlantic, the Gulf of Mexico, the Caribbean [47], and the South Atlantic (Fig 2), but our study was able to confirm its presence only in the Western Atlantic, Bermuda, and the northeastern Gulf of Mexico. We found evidence that samples from farther south in the western Caribbean are in a separate sibling species (see below). The comparable cluster to *P*. *lowei* in the BOLD i.d. cluster tree suggests that the species also occurs off southeastern Canada, Eastern Mexico, and Belize (S1 Fig).

The range of nucleotide differences among samples within *P*. *lowei* is 0–10 out of 4983. Differences from other clades are highest versus *P*. *berndti* (70–99), lowest versus its sibling species (13–20), and have a range of 42–83 versus other species.

***Polymixia* sp. cf. *P*. *lowei***, the newly indicated sibling species to *P*. *lowei*, is represented by samples from Caribbean waters off Panama, but some of the samples in the corresponding cluster of the BOLD i.d. tree (S1 Fig) have labels indicating waters off U.S.A. Unfortunately, the data to support those other locations are not public. The sibling species are phylogenetically distinct (reciprocally monophyletic) and there are no known genetic intermediates. Similar distinctiveness is seen in the BOLD i.d. tree for COI (S1 Fig).

Samples of this previously unrecognized species cluster differ among themselves by only 1–5 site differences in our data. Our results also show that one specimen originally identified as *P*. *japonica* belongs to *P*. sp. cf. *P*. *lowei*. Similarly, the corresponding cluster in the BOLD i.d. tree contains one sample that had been identified as *P*. *nobilis*. The genetic evidence thus highlights this clade as meriting further study and formally named species status.

#### *Polymixia nobilis* species group

This species group contains the type species of the genus, *Polymixia nobilis*, and its likely junior synonym *P*. *busakhini*, along with a different and possibly new species here termed *Polymixia* sp. cf. *P*. *nobilis*.

***Polymixia nobilis*** is recognized here by specimens from the type locality of Madeira in the eastern North Atlantic. A single sample from the MNHN (Paris) thought to be of *P*. *nobilis* but without locality data is here confirmed as being correctly identified. Surprisingly, the *P*. *nobilis* clade also includes the single specimen (originally two samples but with identical sequence) from the submarine Hancock Seamount in the mid-western North Pacific; this specimen had originally been identified as *P*. *japonica* (see above).

***Polymixia busakhini*** was the original identification of two specimens from off Eastern Australia. They are resolved herein within the *P*. *nobilis* clade and are both remarkably similar genetically to those of *P*. *nobilis* from its type locality of Madeira in the NE Atlantic (only 4–5 sequence differences; Fig 7). The two samples of *P*. *busakhini* must therefore be identified as *P*. *nobilis*. Our results thus suggest that *P*. *busakhini* is a junior synonym of *P*. *nobilis*. Unfortunately, no genetic sample of *Polymixia* was available from the area of the type locality of *P*. *busakhini*, a submarine ridge 800 km S of Madagascar [60]. However, Kotlyar also based his description on paratype material from off Eastern Australia, very close to where the vouchers for our two samples identified as *P*. *busakhini* were collected. We note also that morphological characters of *P*. *busakhini* based on the type material in its original description [60] are extremely similar to those of *P*. *nobilis* except for a lower count of pyloric caeca in the former and possibly a subtle difference in shape of the preopercular margin (see below for details).

The cluster that corresponds to *P*. *nobilis* in the BOLD i.d. tree (S1 Fig) does not contain any samples identified as *P*. *nobilis*. However, it does contain samples identified as *P*. *busakhini* or as *P*. *japonica* from Australia, China, and Taiwan. Also included are the same two samples (from a single specimen) discussed above from Hancock Seamount. All of these samples are re-identified here as *P*. *nobilis*. Four additional samples from New Zealand were originally labeled in BOLD as *Polymixia* sp. Those four also can now be identified as *P*. *nobilis*, which had not been identified previously from New Zealand, although it seems likely that the specimens identified as “*Polymixia* cf. *busakhini*” by Roberts [52] are also *P*. *nobilis* and perhaps correspond to some of the BOLD samples from that area.

Sequence diversity within *P*. *nobilis* is remarkably small considering the great geographic distances involved: just 0–5 differences among samples from Madeira in the eastern North Atlantic and those from the southwest Pacific in our data. Sequence differences are large versus samples of other species: 58–84 versus most species and 89–103 versus *P*. *berndti*, but 20–24 versus the single sample here termed *Polymixia* sp. cf. *P*. *nobilis*, discussed below.

*Polymixia nobilis* is now shown to have an antipodal distribution, being known from Madeira in the eastern North Atlantic and near its antipode between Australia and New Zealand. If *P*. *busakhini* from its type area south of Madagascar is confirmed as a junior synonym, *P*. *nobilis* will be shown to have a near worldwide marine distribution at tropical and subtropical latitudes.

***Polymixia* sp. cf. *P*. *nobilis*** is represented by a single specimen originally identified as *P*. *berndti* from off Amami Island in the southern Japanese Archipelago. It is recovered as the sister group of *P*. *nobilis* in all of our analyses. Unfortunately, a sample originally identified as *P*. *sazonovi* from off the nearby island of Okinawa yielded no useful sequence. For the Amami Island specimen, sequence differences are 84–92 versus *P*. *berndti*, 47–58 versus most species, and 20–24 versus *P*. *nobilis*. There is no comparable cluster in the BOLD i.d. tree. This single sample is not part of *P*. *nobilis* because samples of the latter differ among themselves at only 0–5 sites, even when collected from different oceans. The branches leading to this sample and to *P*. *nobilis* are long and support values for the separation of the two are strong (Figs 4–6).

At present it is not entirely clear whether this specimen represents a new species or possibly one of the species previously named but not available to us as genetic samples. Among those are several from more distant locations (*P*. *fusca* from the Arabian Gulf region, *P*. *salagomeziensis* and *P*. *yuri* from SE Pacific seamounts west of Chile; Fig 2). However, the closest geographically is *P*. *sazonovi*, which was named from specimens collected over a submarine ridge about 650 km SE of Amami Island. Morphological features of *P*. *sazonovi* as described by Kotlyar [12] are mostly strikingly similar to those of *P*. *nobilis*. Thus *P*. *sazonovi* should be considered either as a possible identification for our sample of *Polymixia* sp. cf. *P*. *nobilis*, i.e., a distinct species but not a new one, or as a junior synonym of *P*. *nobilis* itself, in which case *Polymixia* cf. *P*. *nobilis* would most likely represent a new species.

**Fig 8:**
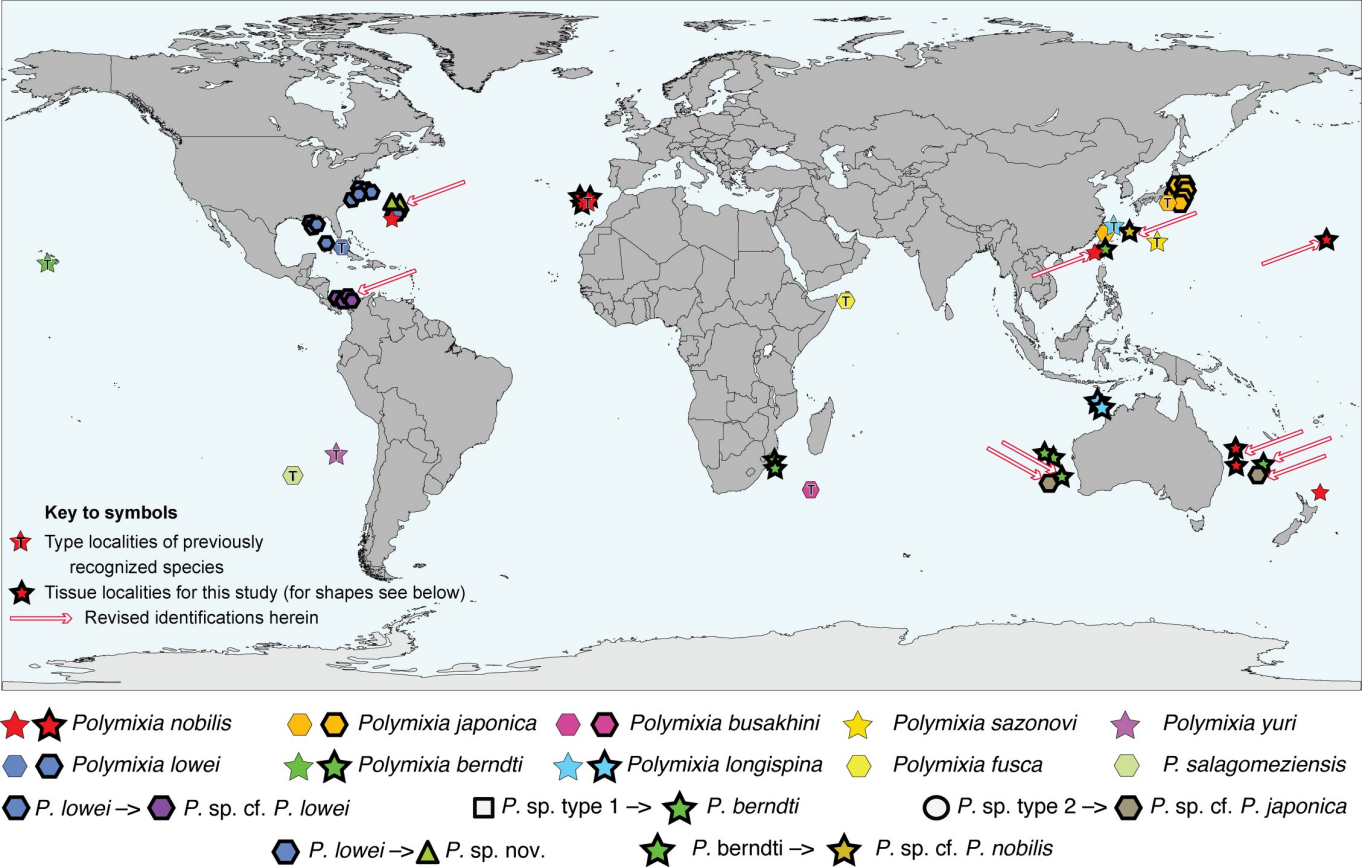
Revised distributions and corrected identifications of species of *Polymixia* based on this study. Compare with Fig 2, which mapped records from museum catalogs and earlier studies. Type localities of species are indicated by symbols with an enclosed letter “T”. DNA sample localities for this study are indicated by symbols with bold outlines; see S2 Table for sample details. Global base map as modified by F. Bennet, in the public domain, accessed at https://commons.wikimedia.org/wiki/File:BlankMap-FlatWorld6.svg.

The Bayesian phylogeny of Fig 4 is shown with corrected species identifications as S2 Fig. Identifications and geographic distributions that are changed by our results are shown in Fig 8 for comparison with Fig 2 (above).

### Meristic and morphological comparisons

Species of *Polymixia* are extremely difficult to distinguish on the basis of meristic and morphological characters. Morphological differences among species are few, subtle, and often variable. Most meristic characters are either identical among species or overlap to such a degree that divisions among species become arbitrary (S1 Table).

**Fig 9:**
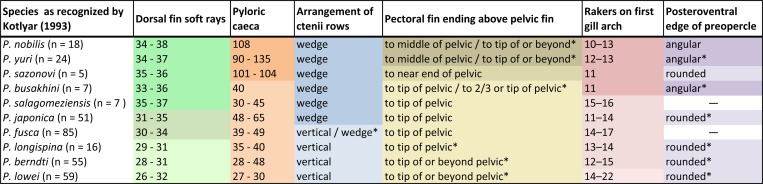
Morphological and meristic characters considered important by Kotlyar. Data are from Kotlyar [12] except those indicated by "*" which are from this study. "—" = data unavailable.

Kotlyar [12] grouped the species of *Polymixia* on the basis of certain characters that he thought most significant (Fig 9). According to him, *Polymixia nobilis*, *P*. *busakhini*, *P*. *salagomeziensis*, and *P*. *sazonovi* share a high dorsal-fin-ray count (34–38). *P*. *berndti*, *P*. *longispina*, and *P*. *lowei* have low dorsal-fin-ray counts (26–32), whereas *P*. *japonica* and *P*. *fusca* have intermediate counts (30–34). A pyloric caeca count of over 100 was used to group *P*. *nobilis*, *P*. *yuri*, and *P*. *sazonovi*, as compared with 65 or fewer in other species of *Polymixia*. In addition, Kotlyar [12] recognized two distinct scale types. *Polymixia nobilis*, *P*. *busakhini*, *P*. *japonica*, *P*. *salagomeziensis*, *P*. *sazonovi*, and *P*. *yuri* have scales with the ctenii arranged in 3–8 wedge shaped rows (apex directed anteriorly) depending on the species. However, he stated that in *P*. *berndti*, *P*. *fusca*, *P*. *longispina*, and *P*. *lowei* the ctenii are arranged in a straight, vertical, marginal band often of one or two rows. Based on scales figured by Kotthaus [54] we would place *P*. *fusca* in the “wedge-shaped” category rather than the “vertical” category (Fig 9).

**Fig 10:**
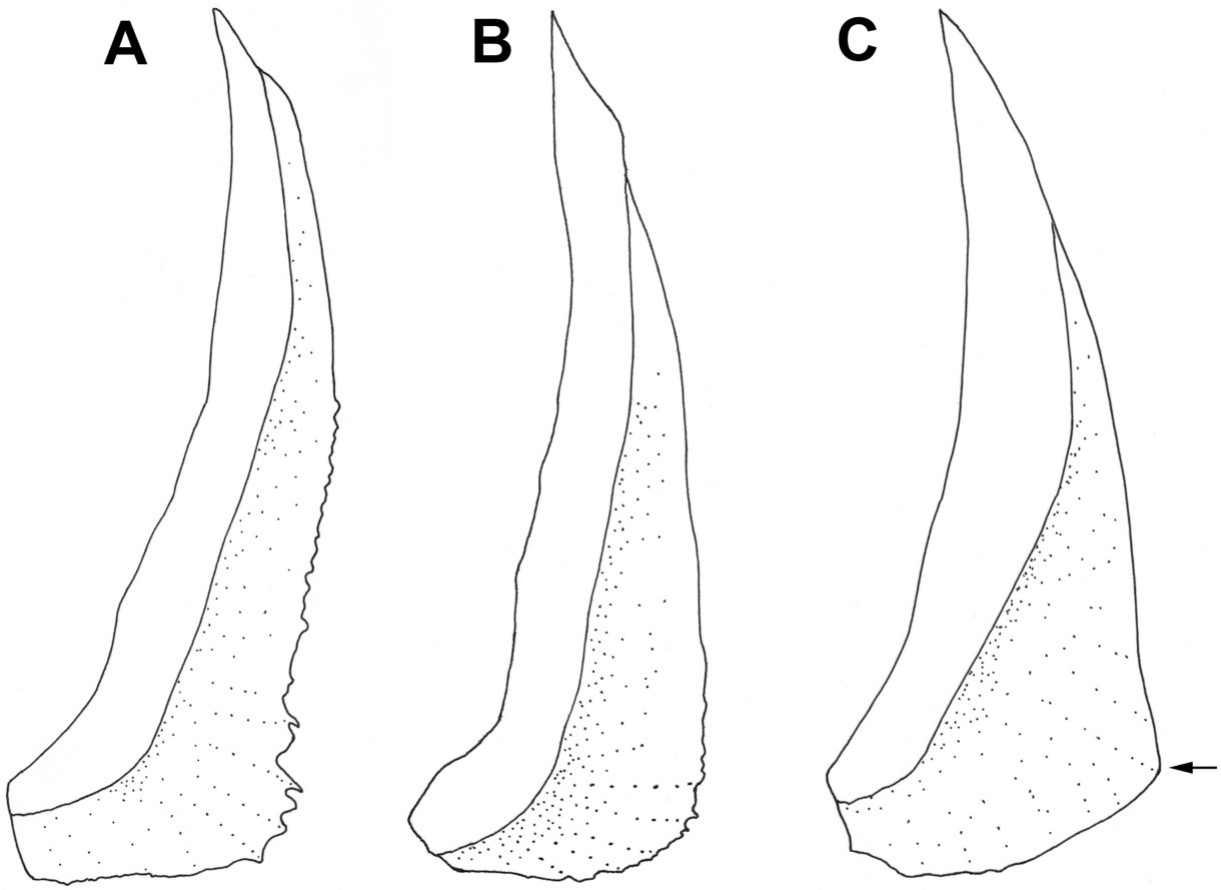
Comparison of preopercle shape in *Polymixia*. A, *P*. *lowei* (UF 44346, 82.4 mm SL) with shallow indentation in lower posterior margin; B, small *P*. *nobilis* (FMNH 64695, 100 mm SL); C, large *P*. *nobilis* (78251, 255 mm SL), with arrow indicating the more angular posteroventral corner seen in larger *P*. *nobilis* of this study.

As per Kotlyar [36], the outer edge of the preopercle in most species of *Polymixia* is rounded, often with a notch or indentation along the posterior margin. Kotlyar [12], however, stated that *P*. *nobilis* differs from all other species in that the preopercle extends posteroventrally to a sharp point. We agree partly with Kotlyar’s assessment, but found that in our specimens of *P*. *nobilis*, the preopercle shape is variable and changes with growth (Fig 10B and 10C). In larger specimens the lower margin is not so sharply pointed but, unlike that of other species, it is more angular and projects slightly farther posteriorly (Fig 10C). The adult condition seen in *P*. *nobilis* was also seen in our single specimen of *P*. *yuri*. We could not check the condition in *P*. *sazonovi*. We also found that the indentation along the lower posterior edge of the vertical limb that is characteristic of most other polymixiids only occurs in specimens over about 80 mm SL (Fig 10A). However, such an indentation was not seen in either the smaller or the larger specimens of *P*. *nobilis* that we examined (Fig 10B and 10C).

Kotlyar [12] also examined the length of the pectoral fin relative to the pelvic fin in species of *Polymixia*. He stated that the pectoral fin in *P*. *nobilis* and *P*. *yur*i extends to a point above the middle of the pelvic fin, that in *P*. *sazonovi* extends to a point above three-fourths the length of the pelvic fins, and the pectoral fin in *P*. *busakhini*, *P*. *japonica*, and *P*. *salagomezensis* extends to a point above the posterior tip of the pelvic fin. We agree that in *P*. *japonica* the pectoral fins are at least as long as the posterior tip of the pelvics, but we have also found this condition in examined specimens of *P*. *berndti*, *P*. *lowei*, *P*. *nobilis*, and *P*. *yuri*. We conclude that this character is not reliable for species discrimination.

A black spot on the soft rays of the dorsal fin was described by Kotlyar [12] in *P*. *sazonovi* and has been reported in other species as well. We were not able to confirm any differences among species for this character in our preserved material, nor were we able to confirm differences that he reported between *P*. *sazonovi* and *P*. *nobilis* in eye diameter or length of the fourth anal-fin spine. In general, we have found the low numbers of individuals of many species available for study, the loss of pigmentation during preservation and storage, and the unknown effects of allometric growth to be problematic. For example, the distance between the tip of the barbels and the base of the pelvic fins used by Kotlyar [12] to identify species might be a function of allometric growth; in small specimens the barbels often extend beyond the base of the pelvic fins, but in large specimens of the same species they do not, ending short of the pelvic fin base.

**Fig 11:**
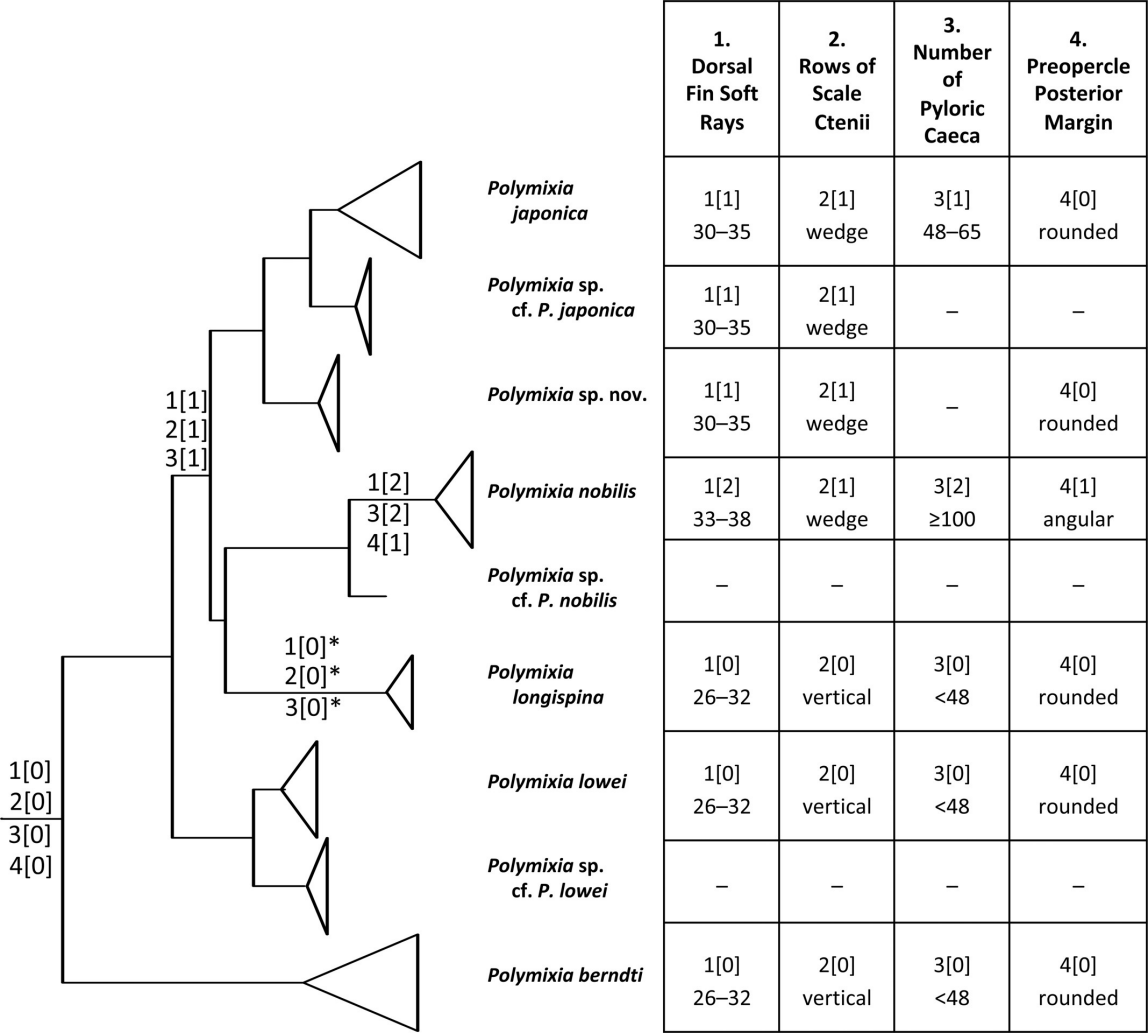
Simplified phylogeny of *Polymixia* with characters mapped. The tree is based on the Bayesian result in Fig 4. Character codes at nodes are as “character(state).” One possible optimization for character-state changes on the *Polymixia longispina* lineage is as reversals, indicated by *. These changes could be explained more parsimoniously if *P*. *longispina* were sister to (*P*. *japonica* species group + *P*. *nobilis* species group), with *P*. *longispina* retaining the primitive states.

Based on our review of the morphological evidence, four characters (number of dorsal-fin soft rays, morphology of rows of scale ctenii, number of pyloric caeca, and shape of lower margin of preopercle), originally proposed by Kotlyar [12], have been identified as being repeatable and possibly having phylogenetic signal. These four characters are plotted by hand on a simplified molecular tree (Fig 11) to illustrate state changes among lineages. Additional taxa not included in the molecular phylogeny are discussed in the text.

1. Number of dorsal-fin rays: 26–32 (0); 30–35 (1); 33–38 (2). *Polymixia longispina*, *P*. *berndti* and *P*. *lowei* share the primitive condition as they have a low number of dorsal-fin rays. *Polymixia japonica* and *P*. *fusca* have an intermediate number of rays, while *P*. *nobilis*, *P*. *yuri*, *P*. *sazonovi*, *P*. *salagomeziensis*, and *P*. *busakhini* share a higher number of fin rays.
2. Morphology of scales anterior to and below the dorsal fin: ctenii arranged in vertical rows (0); ctenii arranged in rows in the shape of a wedge (1). Ctenii arranged in vertical rows were observed in *P*. *lowei*, *P*. *berndti* and reported in *P*. *fusca* and *P*. *longispina*. Ctenii arranged in a wedge pattern (5–6 rows) were observed in *P*. *nobilis*, *P*. *yuri*, *P*. *japonica*, and reported in *P*. *sazonovi*, *P*. *salagomeziensis* and *P*. *busakhini*.
3. Number of pyloric caeca: below 49 (0); 48–65 (1); 100 or more (2). *Polymixia nobilis*, *P*. *yuri* and *P*. *sazonovi* exhibit an extraordinarily high number of pyloric caeca. This high number of pyloric caeca was used to distinguish these three species from the others [12]. *Polymixia japonica* has an intermediate number of caeca while all other species exhibit far fewer.
4. Posterior margin of preopercle: rounded, often with a shallow excavation posteriorly in larger specimens (0), angular and never with an excavation (1). The condition is not reported by Kotlyar [12, 56] for *P*. *fusca* or *P*. *salagomeziensis* and the illustrations of the holotypes in the original descriptions [54, 56] are inconclusive.

With one possible exception, the identified morphological and meristic characters support the relationships as recovered herein, with *P*. *berndti* and *P*. *lowei* retaining primitive character states for several characters, and with the *P*. *japonica* and *P*. *nobilis* species groups being more closely related to each other and sharing some derived morphological character states.

*Polymixia longispina* is an exception. The molecular results give strong support values for including it in a clade with the *P*. *japonica* group and the *P*. *nobilis* group. However, support values are much weaker for placing *P*. *longispina* as more closely related to either the *P*. *japonica* species group or to the *P*. *nobilis* species group. Perhaps future study will show that a third option is correct—*P*. *longispina* sister to both the *P*. *japonica* and the *P*. *nobilis* species groups. *Polymixia longispina* would then be seen as retaining primitive states for all four of the characters mentioned above, whereas the *P*. *japonica* species group and *P*. *nobilis* share the derived state of ctenii in a wedge shape and either intermediate or derived states for dorsal-fin rays and pyloric caeca (Fig 11).

Genetic samples were not available to us for *P*. *fusca*, *P*. *salagomeziensis*, *P*. *sazonovi*, or *P*. *yuri*. However, a single specimen of *P*. *yuri* examined by us, together with Kotlyar’s [55] description, reveals important characters including number of dorsal-fin rays, pyloric caeca, wedge-shaped rows of ctenii, and angular preopercle margin that correspond closely to those of *P*. *nobilis*. *Polymixia sazonovi* as described by Kotlyar [12] is also very similar to *P*. *nobilis*.

Specimens previously identified as *P*. *busakhini* also show morphological characters that are similar to those of *P*. *nobilis*, and genetic samples from those specimens show that they belong in fact to *P*. *nobilis*. Confirmation that *P*. *nobilis* occurs widely in the Pacific makes it plausible that all of these nominal species except for *P*. *fusca* could also be junior synonyms of *P*. *nobilis*.

## Discussion

The present study is novel because it includes the first molecular phylogenetic study of the species of *Polymixia* and also because it employs multiple, geographically documented and vouchered samples for each species when available. This has allowed us to propose a new molecular phylogeny, revise the species composition of *Polymixia*, correct specimen identifications, improve knowledge of geographic distributions, check morphological characters against specimens and, with a phylogeny in hand, to suggest evolutionary transformations for some key characters.

Our results suggest that species richness, taxonomic identifications, and biogeography of species of *Polymixia* are considerably different from the current model. The revised picture changes identifications, confirms the validity of five species, refines geographic distributions of these five species (compare Fig 2 with Fig 8), provides evidence for the junior synonymy of one species (*P*. *busakhini*), and most notably, reveals the existence of up to four previously unrecognized species. The latter four species clades have distinct, monophyletic histories and include a cryptic species (*Polymixia* sp. nov. from Bermuda), a member of an unrecognized sibling species pair (*Polymixia* sp. cf. *P*. *lowei* from off Panama), and two new closest relatives of known species. *Polymixia* sp. cf. *P*. *nobilis*, from near Amami Island, was previously identified as a specimen of *P*. *berndti* but actually represents a new sister species to *P*. *nobilis*. *Polymixia* sp. cf. *P*. *japonica*, from off Eastern and Western Australia, is sister to *P*. *japonica*. It is not truly a cryptic species because Australian experts had recognized both specimens as being unusual morphologically and had provisionally identified them to genus only, as *Polymixia* sp.

Still needed are genetic data for nominal species for which we were not able to obtain samples: *P*. *fusca*, *P*. *salagomeziensis*, *P*. *sazonovi*, and *P*. *yuri*. Whether they are valid and to which species group they are related are important questions for future research. We were able to examine a preserved specimen of *P*. *yuri* and found it to be very similar to *P*. *nobilis*. Further study, including examination of type specimens and discovery of additional specimens, will allow us to test whether *P*. *yuri* and perhaps *P*. *salagomeziensis* and *P*. *sazonovi* are also synonyms of *P*. *nobilis*.

Several species of *Polymixia* have remarkably wide geographic distributions. *Polymixia nobilis* occurs from Madeira in the eastern Atlantic to off Eastern Australia, two locations that are antipodes of each other, yet the individuals are extremely similar genetically. If *P*. *busakhini* is confirmed as a junior synonym, *P*. *nobilis* will be shown to occur also in the western Indian Ocean, giving it a pan-tropical distribution. The *Polymixia berndti* species complex likewise occurs from the Hawaiian Islands to off Mozambique, very nearly antipodal distances apart, although this species complex is genetically more variable than other single species. The *Polymixia japonica* species group, while a clade and not a single species, is represented today from Bermuda to off Western Australia, locations that are also mutually antipodal.

It is challenging to explain such widespread distributions of species and species groups when populations seemingly have patchy local distributions, many of them being recorded only over distal continental shelves, continental slopes, the flanks of oceanic islands, or over the flanks of submarine seamounts. One testable hypothesis might be that dispersal occurs primarily during pelagic phases of early larval ontogenetic stages, about which little is known [88]. With additional life history information, testing this hypothesis will be an important topic for future research.

Nevertheless, this study demonstrates the importance of targeted or genus-level studies to better understand biodiversity. Such revisionary studies, although difficult and time consuming in terms of gathering taxonomically and geographically comprehensive tissues and specimens, are essential for sorting out misidentifications, working out species boundaries, and laying a foundation for higher level, broad-scale studies such as the acanthomorph tree of life and the origins of acanthomorph novelties. As demonstrated in this study, preconceived ideas of species boundaries of *Polymixia* had contributed to misidentifications perpetuated in multiple studies. These include molecular phylogenetic studies [23, 26, 27] using a single specimen of *Polymixia* from the Hancock Seamount in the northwestern Pacific, cited as *P*. *japonica* but actually representing *P*. *nobilis*. The geographic range of *P*. *nobilis* was taken for granted as restricted to the Atlantic Ocean [12, 36, 60]. New evidence from this study, however, suggests that *P*. *nobilis* forms a continuous genetic population, not only in the Atlantic Ocean but also in the Pacific Ocean, and that it occurs in regional sympatry with *P*. *lowei* and *Polymixia* sp. nov. in the Western North Atlantic, as well as with *P*. *berndti*, *P*. *japonica*, and *P*. *longispina* in the Western Pacific.

*Polymixia* is just one example of many genera for which the taxonomic composition was unclear or species boundaries were assumed without thorough study. Recent molecular phylogenetic and morphological studies of fish genera, families, and orders, including this one [89–94], have yielded surprises, including synonyms, misidentified specimens, cryptic and/or sibling species, new genera, unexpected clade memberships, and morphological convergence, thus demonstrating the critical need for such fundamental research.

## Conclusion

Bayesian and maximum-likelihood analyses based on two large fragments of the *16S* mitochondrial DNA gene and five nuclear DNA loci recovered nine species-level clades, five (*Polymixia berndti*, *P*. *japonica*, *P*. *longispina*, *P*. *lowei*, and *P*. *nobilis*) with existing names and four (*Polymixia* sp. cf. *P*. *japonica*, *Polymixia* sp. cf. *P*. *lowei*, *Polymixia* sp. cf. *P*. *nobilis*, and *Polymixia* sp. nov.) likely representing new species.

One nominal species, *Polymixia busakhini*, is likely to be a junior synonym of *P*. *nobilis*. *Polymixia nobilis*, the type species of the genus, is distributed not only in the Atlantic Ocean, but also in the Western and southwestern Pacific, with genetic samples in our study from off Eastern Australia and one specimen from Hancock (submarine) Seamount in the western Hawaiian hot-spot chain. Adding evidence from BOLD, *P*. *nobilis* occurs also in the East China Sea off China and Taiwan, as well as off New Zealand. A single sample from near Amami Island in the southern Japanese Archipelago is the sister group of *P*. *nobilis* in our results. Originally identified as *P*. *berndti*, it represents a distinct species, but which species it is remains in doubt.

*Polymixia japonica* is corroborated as valid but its confirmed distribution is restricted to the areas of Japan, Taiwan, and the East China Sea. Samples from elsewhere identified as *P*. *japonica* or *P*. cf. *P*. *japonica* are re-interpreted as belonging to various other species including *P*. *berndti* (Taiwan), *P*. *nobilis* (Hancock Seamount, Taiwan, China), and *P*. sp. cf. *P*. *lowei* (Caribbean).

Two clades also in the *P*. *japonica* species group both represent new species. One, *Polymixia* sp. cf. *P*. *japonica*, consists of a single sample each from off Eastern and off Western Australia, both formerly identified only to genus as *Polymixia* sp. The other, *Polymixia* sp. nov., is a clade of several samples from waters off Bermuda and is sister to *P*. *japonica* plus *Polymixia* sp. cf. *P*. *japonica*. The as-yet-unnamed Bermuda species, whose formal description is in progress, is a true cryptic species that brings to three the number of species from that area.

*Polymixia berndti* is recovered as the most basal (earliest branching) lineage in *Polymixia* and is also the most distinct as measured by sequence divergence. The large amount of sequence variation among samples within this species complex further suggests that it might in future be divided into more than one species. The *P*. *berndti* species complex ranges from its type locality off the Hawaiian Islands to near Taiwan, off Eastern and Western Australia, and to near Mozambique and South Africa.

*Polymixia longispina* is a relatively rare species with distinctive anal spine morphology originally described from the East China Sea. It has also been recognized off NW Australia. It is not closely related to any of the other species groups and is treated herein as a distinct species group.

*Polymixia lowei* is among the best known and most studied species of *Polymixia*, but our analyses suggest that it should be divided into two sibling species. *Polymixia lowei* was based on a type specimen collected near Cuba and our results include samples or specimens from the northeastern Gulf of Mexico, along the Atlantic coast of the USA, and near Bermuda. Adding records in the BOLD i.d. tree suggests that it can be found also off southeastern Canada, Mexico and Belize. Its sibling species is *Polymixia* sp. cf. *P*. *lowei*, occurring in our samples from off the Caribbean coast of Panama. BOLD data suggest that the sibling species might also occur off the coast of USA, but data to support this extension are not yet public.

Further work is needed to examine *Polymixia* specimens and collect vouchered genetic samples from the type areas of several nominal species. Those efforts together with the revised identifications and geographic distributions presented here will be important in guiding efforts to conserve the taxonomic, genetic, and morphological diversity of this important group of primitive acanthomorph fishes.

## Appendix

Material examined: the following specimens (alcohol preserved, cleared and stained, photos, and/or radiographs) were examined for this study. Data from additional specimens (see S1 Table) were generously provided by J. Pogonoski (CSIRO, Tasmania, Australia).

*Polymixia berndti*: 22 spec., 73–340 mm SL: FMNH 120895 (voucher, alcohol), 120894 (voucher, alcohol), 95583 (alcohol), 120896 (alcohol); NMNH Fin 319433 (photo); USNM 389346 (alcohol, C&S).

*Polymixia lowei*: 67 spec., 65–175 mm SL: ANSP 144889 (alcohol), 105710 (alcohol); BAMZ 1984-047-006, 1989-047-003 (alcohol); KU 30367 (C&S skull only); MCZ 39415 (alcohol), 39186 (C&S), 39770 (alcohol), 45907 (C&S); UF 127145 (alcohol), 36330 (alcohol), 40083 (alcohol), 40263 (alcohol), 44346 (alcohol, C&S), 127151 (C&S), 184751 (C&S); USNM 185284 (alcohol, C&S), 398653 (3 of 4, alcohol), 323212 (photo), 185401 (X-ray).

*Polymixia japonica*: 23 spec., 109–238 mm SL: ANSP 88844 (alcohol), ANSP 90603 (alcohol); FMNH 55422 (alcohol), FMNH 63858 (alcohol), FMNH 63859 (alcohol); FMNH 63860 (alcohol), FMNH 63861 (alcohol), FMNH 120897 (voucher, alcohol); USNM 398535 (voucher, alcohol; this specimen was resolved in our phylogeny as *P*. *berndti*).

*Polymixia nobilis*: 12 spec., 104–300 mm SL: ANSP 124292 (alcohol), 78251 (dry skeleton); FMNH 64695 (alcohol, C&S); UF 231494 (alcohol); USNM 398653 (1 of 4, C&S), USNM RAD 118739–001 (X-ray).

*Polymixia sazonovi*: 1 spec. (photo).

*Polymixia* sp. nov.: BAMZ 1997-159-006 (2, vouchers, alcohol).

*Polymixia yuri*: 1 spec., 175 mm SL: FMNH 96566 (alcohol).

## Supporting information

S1 Table Meristic and morphological characters for species of *Polymixia*. (XLSX)

S2 Table Taxon list, locality information, and voucher, tissue, and GenBank numbers. (XLSX)

S1 Fig BOLD identification tree for *Polymixia* with revised identifications resulting from this study. (PDF)

S2 Fig Bayesian phylogeny of species of *Polymixia* as in Fig 4 but with corrected sample identifications resulting from this study. (PDF)

S1 File Combined sequence matrix of five nuclear DNA and two mitochondrial DNA loci aligned under auto option in MAFFT. The file is in Nexus format.
(NEX)

## References

[1] Davesne D, Friedman M, Barriel V, Lecointre G, Janvier P, Gallut C, et al Fossils illuminate character evolution and relationships of Lampridiformes (Teleostei, Acanthomorpha). Zool J Linn Soc. 2014;172(2): 475–498. 10.1111/zoj.12166

[2] Davesne D, Gallut C, Barriel V, Janvier P, Lecointre G, Otero O. The phylogenetic intrarelationships of spiny-rayed fishes (Acanthomorpha, Teleostei, Actinopterygii): fossil taxa increase the congruence of morphology with molecular data. Frontiers Ecol Evol. 2016;4: 129 10.3389/fevo.2016.00129

[3] Patterson C. 1993. An overview of the early fossil record of acanthomorphs. Bull Marine Sci. 1993;52: 29–59.

[4] Günther A. Report on the deep-sea fishes collected by H.M.S. Challenger during the years 1873–76. Scientific Results of the Voyage of H.M.S. Challenger during the years 1873–76. 1887;Zoology 22: 1–335.

[5] Günther A. Preliminary notes on new fishes collected in Japan during the expedition of H. M. S. Challenger. Ann Mag Nat Hist Ser 4. 1877;20(56): 433–446.

[6] Nelson JS, Grande TC, Wilson MVH. Fishes of the Word Fifth Edition New York: John Wiley & Sons; 2016.

[7] Berg LS. Classification of fishes, both recent and fossil. Proceedings of the Zoological Institute of the USSR Academy of Sciences 1940;5: 87–517. (In Russian with English translation)

[8] Greenwood PH, Rosen DE, Weitzman SH, Myers GS. Phyletic studies of teleostean fishes with a provisional classification of living forms. Bull Amer Mus Nat Hist. 1966;131(4): 339–456.

[9] Woods LP, Sonoda PM. Order Berycomorphi (Beryciformes). Mem Sears Fndn Mar Res. 1973;1(6): 263–396.

[10] Zehren SJ. The comparative osteology and phylogeny of the Beryciformes (Pisces: Teleostei). Evol Monogr. 1979;1: 1–389.

[11] Nelson JS. Fishes of the World 2nd Edition New York: John Wiley & Sons; 1984.

[12] Kotlyar AN. A new species of the genus *Polymixia* (Polymixiidae, Beryciformes) from the Kyushyu-Palau Submarine Ridge and notes on the other members of the genus. J Ichthyol. 1993;33: 30–49. (Originally published in Voprosy Ikhtiologii 1992;32(6): 11–26)

[13] Nelson JS. Fishes of the World. New York: John Wiley & Sons; 1976.

[14] Rosen DE, Patterson C. The structure and relationships of the paracanthopterygian fishes. Bull Amer Mus Nat Hist. 1969;141: 357–474.

[15] Rosen DE. An essay on euteleostean classification. Amer Mus Novit. 1985;2827: 1–57.

[16] Stiassny MLJ. The limits and relationships of the acanthomorph teleosts. J Zool Lond (B). 1986;1: 411–460.

[17] Patterson C, Rosen DE. The Paracanthopterygii revisited: order and disorder. In: Cohen DM, editor: Papers on the Systematics of Gadiform Fishes. Nat Hist Mus Los Angeles Co Sci Ser. 1989;32: 5–36.

[18] Stiassny MLJ, Moore JA. A review of the pelvic girdle of acanthomorph fishes, with comments on hypotheses of acanthomorph interrelationships. Zool J Linn Soc. 1992; 104: 209–242.

[19] Johnson GD, Patterson C. Percomorph phylogeny: a survey of acanthomorphs and a new proposal. Bull Marine Sci. 1993;52: 554–626.

[20] Wiley EO, Johnson GD. A teleost classification based on monophyletic groups In: Nelson JS, Schultzel HP, Wilson MVH, editors. Origin and Phylogenetic Interrelationshipsof Teleosts. Munich: Verlag Pfeil; 2010 pp. 123–182.

[21] Wiley EO, Johnson GD, Dimmick WW. The interrelationships of acanthomorph fishes: a total evidence approach using molecular and morphological data. Biochem Syst Ecol. 2000;28: 319–350. 10725591 10.1016/s0305-1978(99)00069-1

[22] Miya M, Kawaguchi A, Nishida M. Mitogenomic exploration of higher teleostean phylogenies: a case study for moderate-scale evolutionary genomics with 38 newly determined complete mitochondrial DNA sequences. Molec Phylog Evol. 2001;18: 1993–2009. 10.1093/oxfordjournals.molbev.a003741 11606696

[23] Grande T, Borden WC, and Smith WL. Limits and relationships of Paracanthopterygii: A molecular framework for evaluating past morphological hypotheses In: Arratia G, Shultze HP, Wilson MVH, editors. Mesozoic Fishes 5—Global Diversity and Evolution. Munich: Verlag Pfeil 2013 pp. 385–418.

[24] Chen WJ, 2014 New insights on early evolution of spiny-rayed fishes (Teleostei: Acanthomorpha). Front Mar Sci. 2014;1: 1–17.

[25] Santini F, Harmon LJ, Carnevale G, and Alfaro ME. Did genome duplication drive the origin of teleosts? A comparative study of diversification in ray-finned fishes. BMC Evol Biol. 2009;9: 194–209. 10.1186/1471-2148-9-194 19664233 PMC2743667

[26] Betancur R. R, Broughton RE, Wiley EO, Carpenter K, López JA, Li C, et al The tree of life and a new classification of bony fishes. PLOS Currents Tree of Life. 2013; Apr 18 Edition 1, pp. 1–41. 10.1371/currents.tol.53ba26640df0ccaee75bb165c8c26288 23653398 PMC3644299

[27] Near TJ, Dornburg A, Eytan RI, Keck BP, Smith WL, Kuhn KL, et al Phylogeny and tempo of diversification in the superradiation of spiny-rayed fishes. Proc Nat Acad Sci. 2013;110: 12738–12743. 10.1073/pnas.1304661110 23858462 PMC3732986

[28] Miya M, Satoh TP, Nishida M. The phylogenetic position of toadfishes (order Batrachoidiformes) in the higher ray-finned fish as inferred from partitioned Bayesian analysis of 102 whole mitochondrial genome sequences. Biol J Linn Soc. 2005;85: 289–306.

[29] Dettaï A, Lecointre G. Further support for the clades obtained by multiple molecular phylogenies in the acanthomorph bush. C R Biologies. 2005;328: 674–689. 10.1016/j.crvi.2005.04.002 15992750

[30] Miya M, Holcroft NI, Satoh TP, Yamaguchi M, Nishida M, Wiley EO. Mitochondrial genome and a nuclear gene indicate a novel phylogenetic position of deep-sea tube-eye fish (Stylephoridae). Ichthyol Res. 2007;54(4): 323–332.

[31] Miya M, Nishida M. The mitogenomic contributions to molecular phylogenetics and evolution of fishes: a 15-year retrospect. Ichthyol Res. (2014) 2015;62(1): 29–71.

[32] Alfaro ME, Faircloth BC, Harrington RC, Sorenson L, Friedman M, Thacker CE, et al Explosive diversification of marine fishes at the Cretaceous–Palaeogene boundary. Nature Ecol Evol. 2018; 2: 688–696, 10.1038/s41559-018-0494-6 pp. 1–11. 29531346

[33] Hughes LC, Ortí G, Huang Y, Sun Y, Baldwin CC, Thompson AP, et al Comprehensive phylogeny of ray-finned fishes (Actinopterygii) based on transcriptomic and genomic data. Proc Nat Acad Sci. 2018; 10.1073/PNAS.1719358115 29760103 PMC6004478

[34] Borden WC, Grande TC, Smith WL. Comparative osteology and myology of the caudal fin in the Paracanthopterygii (Teleostei: Acanthomorpha). In: Arratia G, Shultze HP, Wilson MVH, editors. Mesozoic Fishes 5—Global Diversity and Evolution. Munich: Verlag Pfeil 2013 pp. 419–455.

[35] Patterson C, Johnson GD. The intermuscular bones and ligaments of teleostean fishes. Smithson Contr Zool. 1995;559: 1–85.

[36] Kotlyar AN. Systematics and the distribution of fishes of the family Polymixiidae (Polymixioidei, Beryciformes). J Ichthyol. 1984;22: 1–20. (Originally published in Voprosy Ikhtiologii. 1984;5: 691–708).

[37] Heemstra PC. Family No. 134: Polymixiidae In: Smith MM, Heemstra PC, editors. Smith's sea fishes. Johannesburg: Macmillan; 1986 p. 432.

[38] Paxton JR, Hoese DF, Allen GR, Hanley JE. Zoological catalogue of Australia. V. 7. Pisces Petromyzontidae to Carangidae. Canberra: Austral Gov Publ Serv 1989.

[39] Starks EC. The osteology of some berycoid fishes. Proc US Nat Mus. 1904;27(1366): 601–619.

[40] Near TJ, Eytan RI, Dornburg A, Kuhn KL, Moore JA, Davis MP, et al Resolution of ray-finned fish phylogeny and timing of diversification. Proc Nat Acad Sci. 2012;109: 13698–13703. 10.1073/pnas.1206625109 22869754 PMC3427055

[41] Tanaka S. Figures and descriptions of the fishes of Japan. 1913;3(82): 218–220.

[42] Kamohara T. Revised descriptions of the offshore bottom-fishes of Prov. Tosa, Shikoku, Japan. Rep Kochi Univ Nat Sci. 1952;3: 1–122.

[43] Lachner EA. Populations of the berycoid fish family Polymixiidae. Proc US Nat Mus. 1955;105(3356): 189–206.

[44] Yamane S, Okamura O. A review of the Pacific species of the berycoid fish belonging to the genus *Polymixia*. Bull Misaki Mar Biol Inst Kyoto Univ. 1966;9: 13–20.

[45] Lowe RT. Piscium Maderensium species quaedam novae, vel minus rite cognitae breviter descriptae, etc. Trans Cambr Philos Soc. 1836;6(1): 195–202.

[46] Günther A. Catalogue of the acanthopterygian fishes in the British Museum Vol. 1 London: British Museum; 1859 pp. 16–19.

[47] Moore J. Order Polymixiiformes, Polymixiidae, Beardfishes In: Carpenter KE editor. The living marine resources of the western central Atlantic. Rome: FAO Species Identification Guide for Fishery Purposes Amer Soc Ich Herp Spec Pub. 2002;5: 960–962.

[48] Moore J, Polanco Fernandez A, Russell B, McEachran JD, Iwamoto T. *Polymixia nobilis*, Stout Beardfish. The IUCN Red List of Threatened Species 2015; e.T15622706A15623397. 10.2305/IUCN.UK.2015-4.RLTS.T15622706A15623397.en Accessed 09 January 2019.

[49] Valenciennes A. Ichthyologie des Iles Canaries ou histoire naturelle des poissons In: Webb PB, Berthelot S, editors. Histoire naturelle des Iles Canaries. Paris: Béthune; 1836–1844;2(2):1–109.

[50] Poey F. Poissons de Cuba. In: Memorias sobre la historia natural de la Isla de Cuba. 1860;2(49): 115–336.

[51] Nichols JT, Firth FE. A new triacanthid fish and other species from deep water off Virginia. Amer Mus Novit. 1936;883: 1–5.

[52] Roberts CD. 95 Family Polymixiidae In: Roberts CD, Stewart AL, Struthers CD, editors. The fishes of New Zealand v.3. Wellington: Te Papa Press; 2015 pp. 698–700.

[53] Gilbert CH. The deep-sea fishes of the Hawaiian lslands. Bull US Fish Comm. 1905;23(2): 578–713.

[54] Kotthaus A. Fische des ichthyologischen Untersuchungen während der Expedition der Forschungsschiffes *Meteor* in den Indischen Ozean, Oktober 1964 bis Mai 1965. A. Systematischer Teil, VI, Anacanthini (2), Berycomorphi, Zeomorphi. Meteor Forsch Ergebnisse. 1970;D(5): 53–70.

[55] Kotlyar AN. *Polymixia yuri* sp. n. (Beryciformes, Polymixiidae) from the southeastern Pacific Ocean. Zool Zhurn. 1982;61(9): 1380–1384.

[56] Kotlyar AN. A new species of the genus *Polymixia* from the Sala y Gomez submarine ridge. Zool Zhurn. 1991;70(7): 83–86.

[57] Deng SM, Xiong GQ, Zhan HX. Two new species of deep sea fishes from the East China Sea. Acta Zootaxon Sinica. 1983;8: 317–322.

[58] Okamura O, Machida Y, Yamakawa T, Matsuura K, Yatou T. Fishes of the Okinawa Trough and the adjacent waters. Vol. 2. The intensive research of unexploited fishery resources on continental slopes. Japan Fish Res Conserv Assoc, Tokyo. 1985;2: 418–781.

[59] Okamura O. Polymixia kawadae Okamura et Ema The fishes of the Japanese Archipelago. Tokyo: Tokai University Press; 1988.

[60] Kotlyar AN. Beryciform fishes from the western Indian Ocean collected in cruise of R/V “Vityaz.” Trans PP Shirshov Inst Oceanol. 1992;128: 179–198.

[61] Kotlyar AN On the biology of *Polymixia berndti* Gilbert (Polymixiidae) in the western part of the Indian Ocean. J Ichthyol. 1986;26(2): 120–127.

[62] Heemstra PC, Fricke H, Hissmann K, Schauer J, Smale M, Sink K. Interactions of fishes with particular reference to coelacanths in the canyons at Sodwana Bay and the St Lucia Marine Protected Area of South Africa. S Afr J Sci. 2006;102: 461–465.

[63] Titus TA. A phylogenetic analysis of the Desmognathinae (Caudata: Plethodontidae): Evolutionary patterns inferred from mitochondrial DNA sequences. Unpubl. Ph.D. Dissertation, University of Kansas. 1992.

[64] Feller AE, Hedges SB. Molecular evidence for the early history of living amphibians. Mol Phylogenet Evol. 1998;9: 509–516. 10.1006/mpev.1998.0500 9667999

[65] Kocher TD, Thomas WK, Meyer A, Edwards SV, Pääbo S, Villablanca XF, et al Dynamics of mitochondrial DNA evolution in animals: amplification and sequencing with conserved primers. Proc Nat Acad Sci. 1989;86: 6196–6200. 2762322 10.1073/pnas.86.16.6196 PMC297804

[66] Palumbi SR. Nucleic acids II: the polymerase chain reaction In: Hillis DM, Moritz C, Mable BK, editors. Molecular Systematics, 2nd ed Sunderland, Massachusetts: Sinauer 1996 pp. 205–247.

[67] Li C, Ortí G, Zhang G, Lu G. A practical approach to phylogenomics: The phylogeny of ray-finned fish (Actinopterygii) as a case study. BMC Evol Biol. 2007;7: 44 10.1186/1471-2148-7-44 17374158 PMC1838417

[68] Holcroft NJ. A molecular test of alternative hypotheses of tetraodontiform (Acanthomorpha: Tetraodontiformes) sister-group relationships using data from the RAG1 gene. Mol Phylog Evol. 2004;32: 749–760. 10.1016/j.ympev.2004.04.002 15288052

[69] Smith WL, Wheeler WC. Polyphyly of the mail-cheeked fishes (Teleostei: Scorpaeniformes): evidence from mitochondrial and nuclear sequence data. Mol Phylog Evol. 2004;32(2): 627–646. 10.1016/j.ympev.2004.02.006 15223043

[70] Satoh T, Miya M, Mabuci K, Nishida M. Structure and variation of the mitochondrial genome of fishes. BMC Genomics. 2016;17: 719 10.1186/s12864-016-3054-y 27604148 PMC5015259

[71] Katoh K, Standley DM. MAFFT multiple sequence alignment software version 7: improvements in performance and usability. Mol Biol Evol. 2013;30: 772–780. 10.1093/molbev/mst010 23329690 PMC3603318

[72] Katoh K, Rozewicki J, Yamada KD. MAFFT online service: multiple sequence alignment, interactive sequence choice and visualization. Briefings in Bioinformatics. 2017; 10.1093/bib/bbx108 28968734 PMC6781576

[73] Lanfear R, Frandsen PB, Wright AM, Senfeld T, Calcott B. PartitionFinder 2: new methods for selecting partitioned models of evolution for molecular and morphological phylogenetic analyses. Mol Biol Evol. 2016;34(3): 772–773. 10.1093/molbev/msw260 28013191

[74] Lanfear R, Calcott B, Ho SY, Guindon S. PartitionFinder: combined selection of partitioning schemes and substitution models for phylogenetic analyses. Mol Biol Evol. 2012;29(6): 1695–1701. 10.1093/molbev/mss020 22319168

[75] Guindon S, Dufayard JF, Lefort V, Anisimova M, Hordijk W, Gascuel O. New algorithms and methods to estimate maximum-likelihood phylogenies: assessing the performance of PhyML 3.0. Syst Biol. 2010;59(3): 307–321. 10.1093/sysbio/syq010 20525638

[76] Ronquist F, Teslenko M, van der Mark P, Ayres D, Darling A, Höhna S, et al MrBayes 3.2: Efficient Bayesian phylogenetic inference and model choice across a large model space. Syst Biol 2012;61(3): 539–542. 10.1093/sysbio/sys029 22357727 PMC3329765

[77] Geyer CJ. Markov chain Monte Carlo maximum likelihood In: Kerimidas EM, editor. Computing Science and Statistics: Proceedings of the 23rd Symposium on the Interface. Fairfax Station: Interface Foundation 1991 pp. 156–163.

[78] Brown JM, Hedtke SM, Lemmon AR, Lemmon EM. 2010. When trees grow too long: investigating the causes of highly inaccurate Bayesian branch-length estimates. Syst Biol. 2010;59: 145–161. 10.1093/sysbio/syp081 20525627

[79] Marshall DC. Cryptic failure of partitioned bayesian phylogenetic analyses: lost in the land of long trees. Syst Biol. 2010;59(1): 108–117. 10.1093/sysbio/syp080 20525623

[80] Rambaut A, Suchard MA, Xie W, Drummond AJ. Tracer v1.6.1. 2014 Available from: http://tree.bio.ed.ac.uk/software/tracer/

[81] Rambaut A. FigTree: Tree Figure Drawing Tool. v1.4.3. 2016; Available from: http://tree.bio.ed.ac.uk/software/figtree/

[82] Zwickl DJ. Genetic algorithm approaches for the phylogenetic analysis of large biological sequence datasets under the maximum likelihood criterion. Ph.D. dissertation, The University of Texas at Austin. 2006. Available at: http://www.zo.utexas.edu/faculty/antisense/zwicklDissertation.pdf

[83] Zwickl DJ. Garli v. 2.0. 2011 Available at: https://code.google.com/archive/p/garli/downloads.

[84] Stamatakis A. RAxML Version 8: a tool for phylogenetic analysis and post-analysis of large phylogenies. Bioinformatics. 2014;30(9): 1312–1313. 10.1093/bioinformatics/btu033 Code available from: https://github.com/stamatak/standard-RAxML 24451623 PMC3998144

[85] Cracraft J. Species concepts and speciation analysis. Curr Ornithol. 1983;1: 159–187.

[86] Ratnasingham S, Hebert PDN. 2007. BOLD: The Barcode of Life data system (www.barcodinglife.org). Mol Ecol Notes. 2007;7: 355–364. 10.1111/j.1471-8286.2007.01678.x 18784790 PMC1890991

[87] Smith-Vaniz WF, Collette BB, Luckhurst BE. Fishes of Bermuda: history, zoogeography, annotated checklist, and identification keys Lawrence KS: Amer Soc Ichthyol Herpetol; 1999.

[88] Lyczkowski-Shultz J. Polymixiidae: Beardfishes In: Richards WJ, editor. Early stages of Atlantic fishes. An identification guide for the western central North Atlantic. Boca Raton: CRC Press; 2005 pp. 1105–1107.

[89] Carreras-Carbonell J, Macpherson E, Pascual M. Rapid radiation and cryptic speciation in mediterranean triplefin blennies (Pisces: Tripterygiidae) combining multiple genes. Mol Phyloget Evol. 2005;37: 751–761. 10.1016/j.ympev.2005.04.021 15964768

[90] Lin HC, Hastings PA. Phylogeny and biogeography of a shallow water fish clade (Teleostei: Blenniiformes). BMC Evol Biol. 2013;13(210): 1–18. 10.1186/1471-2148-13-210 24067147 PMC3849733

[91] McMahan CD, Matamoros WA, Piller KL, Chakrabarty P. Taxonomy and systematics of the herichthyins (Cichlidae: Tribe Heroini), with the descriptions of eight new Middle American genera. Zootaxa. 2015;3999(2)L211–234. 10.11646/zootaxa.3999.2.3 26623572

[92] Burns MD, Chatfield M, Birindelli JLO, Sidlauskas BL. Systematic assessment of the *Leporinus desmotes* species complex, with a description of two new species. Neotrop Ichthyol. 2017;15(2): e160166:1–23. 10.1590/1982-0224-20160166

[93] Grande TC, Borden WC, Wilson MVH, Scarpitta L. Phylogenetic relationships among fishes in the order Zeiformes based on molecular and morphological data. Copeia. 2018;106(1): 20–48.

[94] Kai Y, Tashiro F. *Zenopsis filamentosa* (Zeidae), a new mirror dory from the western Pacific Ocean, with redescription of *Zenopsis nebulosa*. Ichthyol Res. 2019 1 12; 10.1007/s10228-018-00679-1

