# Supplementary figures and images for: Phylogenetic relationships within the primitive acanthomorph fish genus Polymixia, with changes to species composition and geographic distributions
Source: PLoS One. 2019 Mar 1;14(3):e0212954. doi: 10.1371/journal.pone.0212954 (PMC6396927; doi:10.1371/journal.pone.0212954)

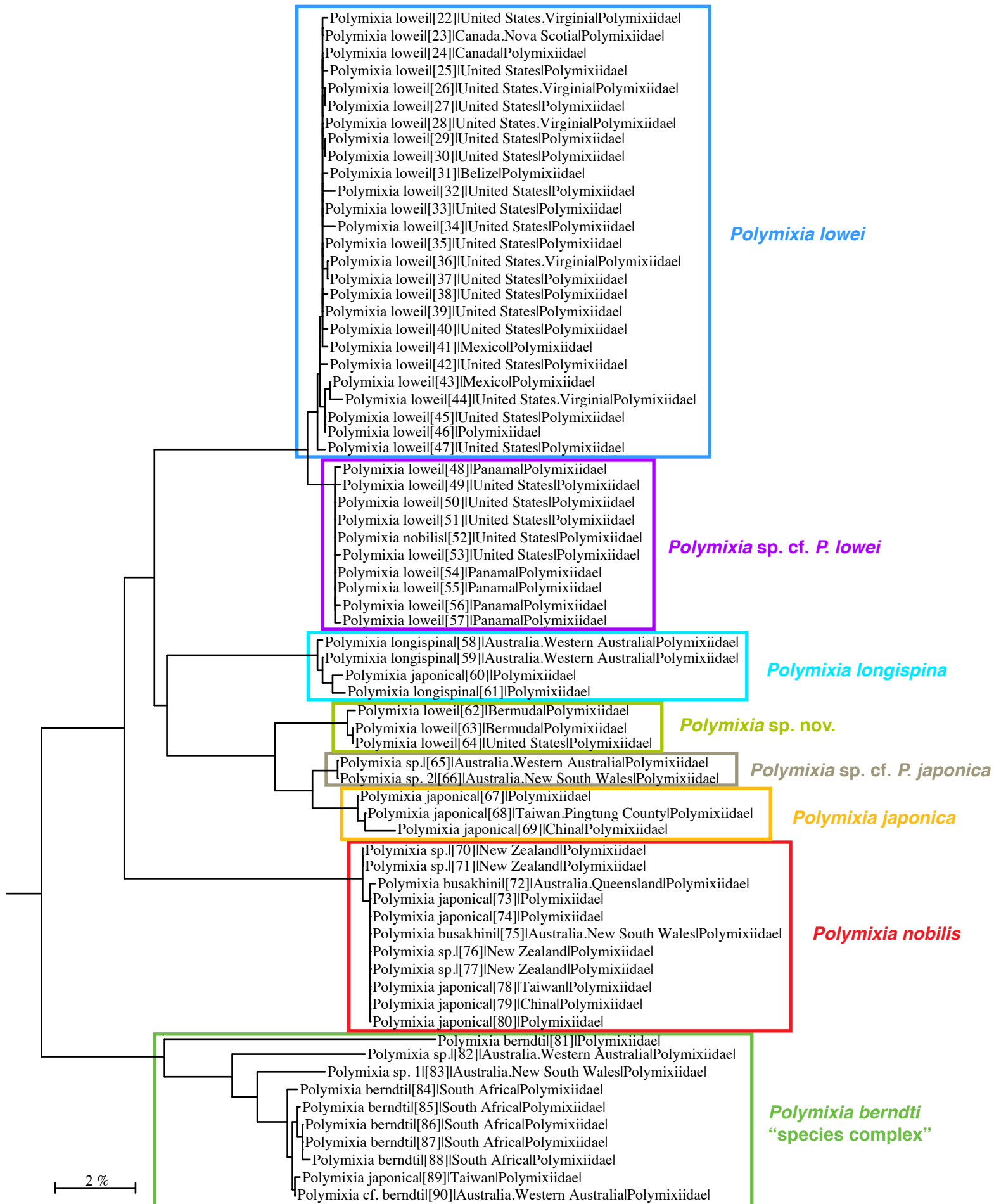

Supplement: S1 Fig — (PDF) [file pone.0212954.s003.pdf]

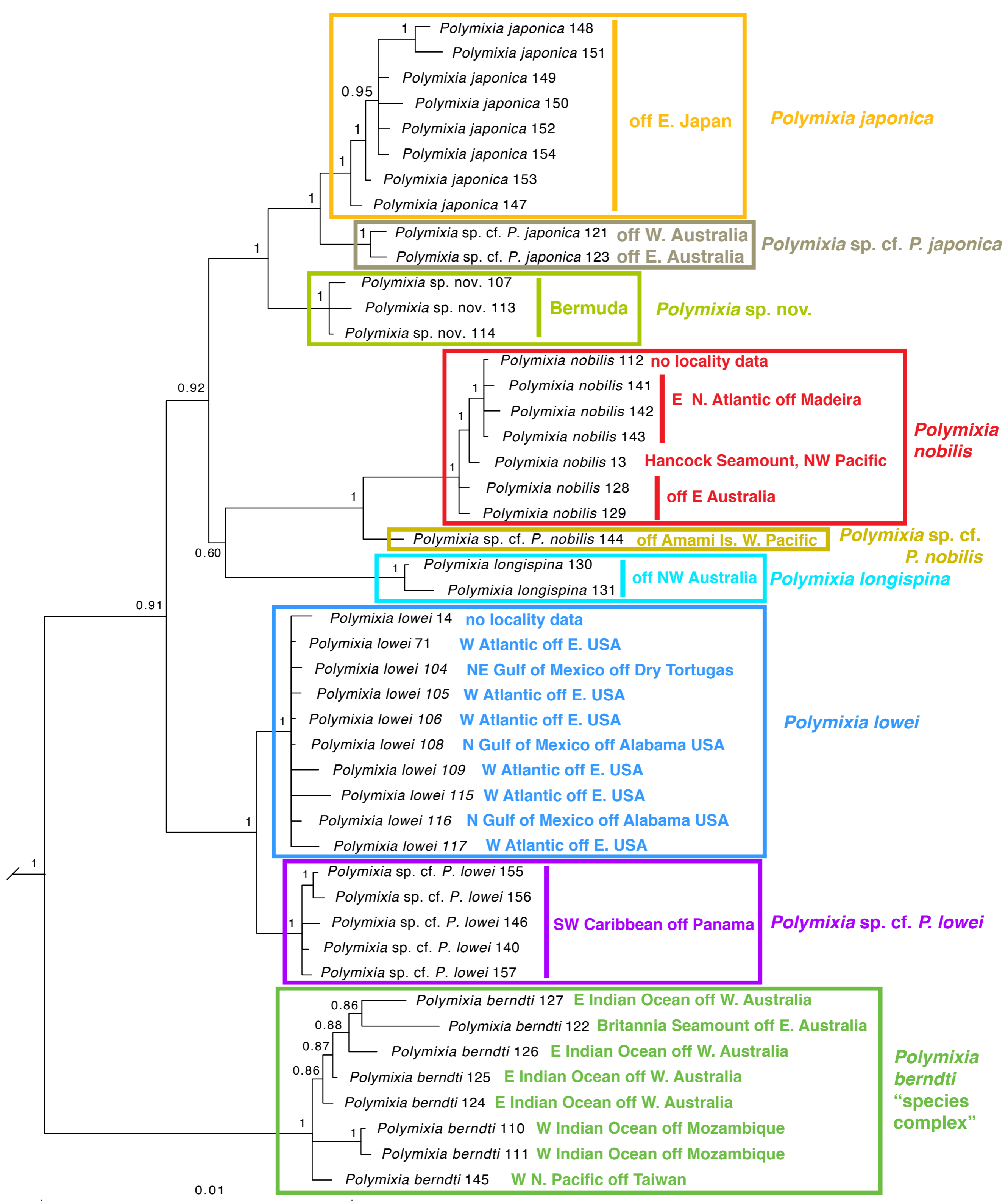

Supplement: S2 Fig — (PDF) [file pone.0212954.s004.pdf]
